# Nanomaterials based on thermosensitive polymer in biomedical field

**DOI:** 10.3389/fchem.2022.946183

**Published:** 2022-09-21

**Authors:** Yingshu Guo, Li Sun, Yajing Wang, Qianqian Wang, Dan Jing, Shiwei Liu

**Affiliations:** ^1^ Shandong Provincial Key Laboratory of Molecular Engineering, School of Chemistry and Chemical Engineering, Qilu University of Technology (Shandong Academy of Sciences), Jinan, China; ^2^ School of Chemistry and Chemical Engineering, Linyi University, Linyi, China

**Keywords:** temperature sensitive, nanomaterials, sol–gel transition, polymer, bioanalysis

## Abstract

The progress of nanotechnology enables us to make use of the special properties of materials on the nanoscale and open up many new fields of biomedical research. Among them, thermosensitive nanomaterials stand out in many biomedical fields because of their “intelligent” behavior in response to temperature changes. However, this article mainly reviews the research progress of thermosensitive nanomaterials, which are popular in biomedical applications in recent years. Here, we simply classify the thermally responsive nanomaterials according to the types of polymers, focusing on the mechanisms of action and their advantages and potential. Finally, we deeply investigate the applications of thermosensitive nanomaterials in drug delivery, tissue engineering, sensing analysis, cell culture, 3D printing, and other fields and probe the current challenges and future development prospects of thermosensitive nanomaterials.

## 1 Introduction

Over the past 2 decades, the biomedical field has experienced unprecedented advances in the development of novel nanomaterials. Among various nanomaterials, the ability to respond to environmental stimuli has attracted more attention from researchers to new “smart” responsive nanomaterials (Sanchez-Moreno et al., 2018; Pham et al., 2020). The smart responsive nanomaterial is an intelligent material capable of sensing changes in external or internal environment and effectuating a rapid response (Vanparijs et al., 2017). Smart responsive nanomaterials respond to external or internal environmental stimuli, such as changes in the temperature, pH, light, electric field, magnetic field, enzyme, and redox substances by altering their own physical and chemical properties or inducing structural changes (Norouzi et al., 2016; Zhang K. et al., 2018; Doberenz et al., 2020; Pham et al., 2020). The extensive development of intelligent nanomaterials has major potential in the medical field. In particular, intelligent nanomaterials that respond to temperature changes are among the most widely used exogenous stimulus response systems (Pham et al., 2020). Thermosensitive polymers, as a subclass of smart polymers, undergo a temperature-induced reversible sol–gel transition when polymer aqueous solutions are heated or cooled (El-Zaafarany et al., 2018). The design of thermosensitive nanomaterials with a good temperature response and their ability to exhibit coil to ball transition behavior when approaching physiological temperature, which make them more promising materials in the field of biomaterials and biomedicine (Narang and Venkatesu, 2019). Therefore, this review focuses on thermosensitive nanomaterials.

Polymers that sense changes in ambient temperature and alter their solubility accordingly are referred to as thermosensitive polymers. Usually, we regard the characteristic of temperature response as the ability of the polymer to dissolve in an aqueous medium (Bordat et al., 2019). The response of such thermosensitive polymers to stimuli is usually accompanied by conformational changes in their polymer structures. The temperature at which the polymer structure or solubility changes is referred as the phase transition temperature or critical solution temperature (Karimi et al., 2016; Sponchioni et al., 2019). The solubility of the aqueous solutions of some polymers decreases with an increase in the temperature. Upon reaching a specific temperature, the solutions become turbid due to phase separation. Below this temperature, they become a clear and transparent solution again. At this time, the temperature is the low critical solution temperature (LCST). On the contrary, other polymers only dissolve above a certain temperature, which is referred to as the high critical solution temperature (UCST) (Le et al., 2018; Sponchioni et al., 2019). Therefore, according to different solution temperatures, thermosensitive polymers can be divided into polymers with low and high critical dissolution temperatures. Currently, thermosensitive polymers with low critical solution temperature are widely studied and applied. Hence, this review focuses on thermosensitive polymers with low critical solution temperature.

**FIGURE 1:**
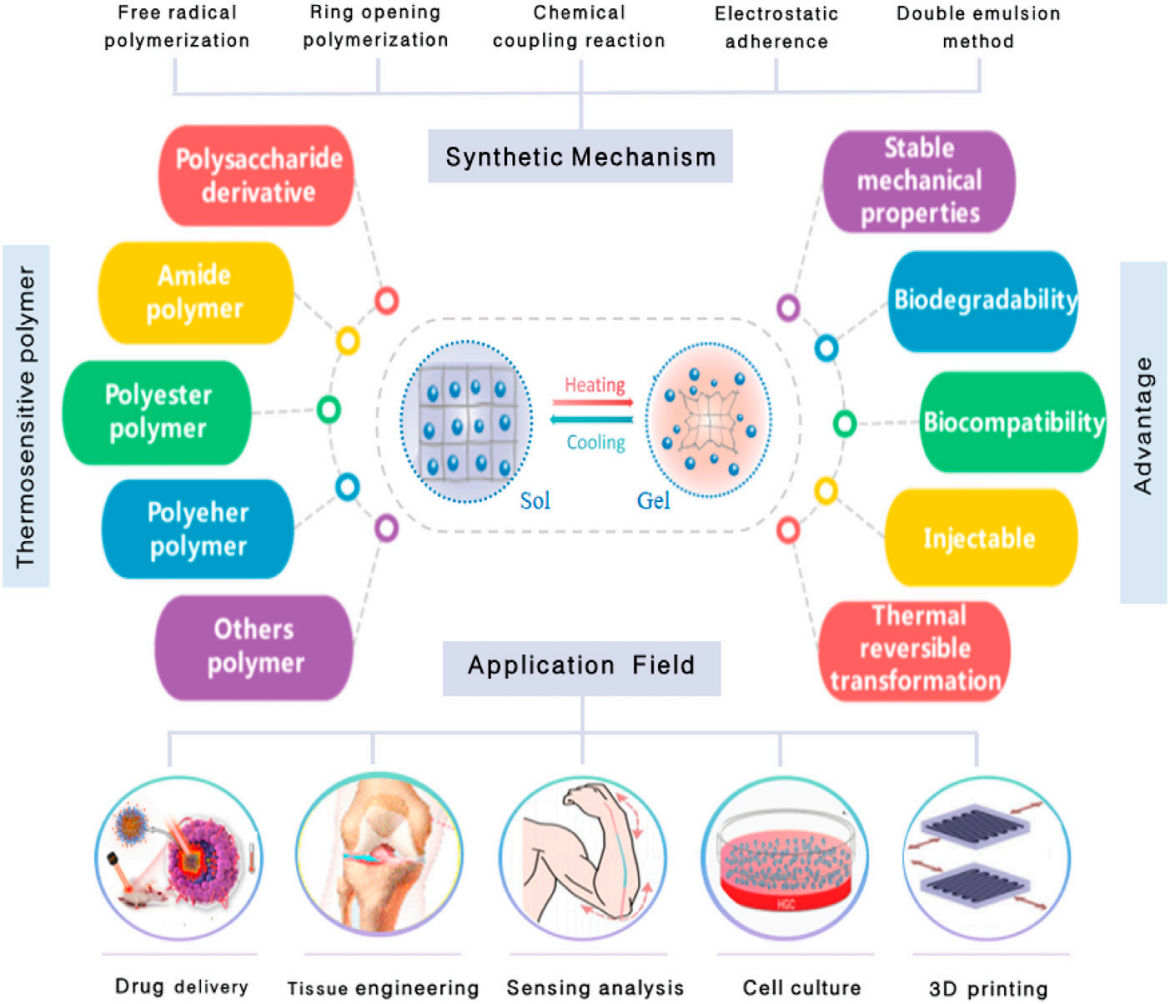
Synthetic mechanism, types, properties of thermosensitive nanomaterials, and their applications in the biomedicine field.

Owing to its unique temperature sensitive characteristics, the nanomaterial based on thermosensitive polymers has been widely used as a new biomaterial in biomedicine and other fields (Sun et al., 2020). Most nanomaterials based on thermosensitive polymers consist of a macromolecular backbone, hydrophilic, and hydrophobic groups. When the external temperature changes, the intramolecular and intermolecular interactions between hydrophilic and hydrophobic groups and water molecules change, which affect the internal network structure of the polymer and cause volume phase transition (Vanparijs et al., 2017). The mechanism of the response to temperature stimulation is as follows: at a lower temperature, hydrophilic groups and water molecules are bonded by hydrogen bonds to form a solution. With the increase of temperature, the interaction forces between polymer and water molecules weaken. However, the force of association between hydrophobic groups dominates, leading to polymer aggregation and phase transformation (Sanchez-Moreno et al., 2018; Sponchioni et al., 2019). On the other hand, the phase transition temperature of the thermosensitive nanomaterial applied in biomedical research fields is close to the physiological temperature of the human body, so that it exhibits the advantages of good biocompatibility, injectable, and so on. The thermosensitive nanomaterial not only plays an important role in drug delivery but also holds great significance in biological sensing, biomimetic medicine, tissue engineering, and so on (Figure 1) (Pham et al., 2020).

Because temperature is an easily applicable stimulus, it can be used as an external stimulus when heated from the outside or as an internal stimulus when the temperature of pathological lesions increases naturally (Sponchioni et al., 2019). The thermosensitive nanomaterial has become one of the most important intelligent nanomaterials studied in recent decades. Hence, recent research works and applications of thermosensitive nanomaterials are reviewed in this study. Specifically, we focus on the types, properties, and action mechanisms of thermal response nanomaterials and their applications in the biomedical field. In addition, we further comprehensively retrospected their challenges in current biomedical applications and their future applications.

## 2 Types of thermosensitive nanomaterials

The thermosensitive polymer is a kind of polymer that undergoes phase change with changes in the ambient temperature. According to different sources, it can be segmented into natural and synthetic polymers (Bellotti et al., 2021). Among them, most natural polymers are polysaccharide derivatives. While the internal structure of synthesized polymers often contains substituted amides, ether bonds, hydroxyl groups, and other functional groups, which are easily functionalized and modified (Hajebi et al., 2019). Since the thermosensitive nanomaterial is based on thermosensitive polymer, its classification also refers to the type of thermosensitive polymer. Common types of nanomaterials based on thermosensitive polymers are as follows:

### 2.1 Polysaccharide

#### 2.1.1 Chitosan

Chitosan (CS) is a natural amino polysaccharide, which is the product of chitin N-deacetylation. It has numerous physiological functions, such as biodegradability, biocompatibility, and bacteriostasis (Zhang et al., 2019). The chitosan molecule simultaneously contains amino, acetyl, and hydroxyl groups, which makes it with excellent biological functions and capability to be chemically modified (Ali and Ahmed, 2018).

CS itself is a natural polymer without temperature sensitivity, but it can gain this function upon modification by various chemical functions. The solution is a flowable liquid at room temperature and can be easily injected into the body. In addition, the existence of β-glycerophosphate (β-GP) makes CS solution gel automatically at physiological temperature. This is due to the enhancement of hydrogen bonds, electrostatic attractions, and hydrophobic interactions between CS and β-GP. The rheological properties of the CS/β-GP thermosensitive nanomaterial showed that the nanomaterial is a stable viscous liquid below 29°C, and the sol–gel transition temperature is between 29 and 37°C (Mohammed et al., 2017). It was found that the nanomaterial completely gels at 37°C in only about 3.3 min, which would be beneficial to minimally invasive injection *in vivo*. Notably, the gel state of the nanomaterial has not changed after being heated to 60°C, indicating that the gelation process is irreversible (Zheng et al., 2019). Song et al. observed that the surface of the CS/β-GP nanomaterial was porous and spongy, and the pores were loose, irregular, and large. However, the addition of collagen (Col) not only reduces the cytotoxicity of β-GP but also enhances the stability of the nanomaterial structure. This leads to the polymerization of CS, such that the morphology of the nanomaterial is characterized by dense, uniform, and coherent porous inner connecting tubes. From the curves of rheological analysis, we can observe that the gelation temperature is in the range of 36–38°C (Table 1) (Song et al., 2017). Moreover, Shan et al. pointed out that the CS/β-GP/Col thermosensitive nanomaterial could be converted from solution to gel within 60–80 s at physiological temperature, as the higher deacetylated CS could shorten the gel time. Undeniably, compared with the 90% CS, the nanomaterial containing 95% deacetylated CS has better physical and chemical properties (Shan et al., 2019). On this basis, Tang et al. further studied the effect of the addition of hydroxypropyl cellulose (HPC) on its physical and chemical properties. It was found that the addition of HPC shortened the gel time, which may be caused by the increased interaction between macromolecules. Simultaneously, HPC can reduce the gel temperature and maintain the gel temperature between 16 and 19°C. Moreover, the addition of HPC significantly improved the viscosity, strength, and biological adhesion of the gel (Tang et al., 2019). Notably, Wang et al. proposed that grafted CS with β-cyclodextrin (β-CD) to obtain three-dimensional porous CS-g-β-CD with high hydrophilicity. As expected, CS-g-β-CD exhibited significantly enhanced hydrophilicity, thermal stability, and increased specific surface area, and the gelation temperature increased to 37°C, which may be attributed to the three-dimensional porous network structure in CS-g-β-CD (Wang et al., 2019).

**TABLE 1:** List of thermosensitive nanomaterials based on polysaccharide.

Thermosensitive nanomaterial	Synthetic mechanism	LCST	Gel time	Superiority	Application field	Reference
CS/β-GP	Hydrogen bonding, electrostatic attraction, and hydrophobic action	29–37°C	3.3 min	High biological stabiliy and irreversible gel	Antibacterial eye ophthalmic dressings and on-demand drug release	Mohammed et al. (2017)
CS/GP/Col	Hydrogen bonding, electrostatic attraction, and hydrophobic action	36–38°C	60–80 s	High porosity, high degradation rate, and rapid gelation	ADSCs culture *in vitro* and treat ulcers	Song et al., 2017 Shan et al. (2019)
CS/HPC/Col-GP	Hydrogen bonding, electrostatic attraction, and hydrophobic action	16–19°C	2min	Good biocompatibility	Endoscopic mucosal dissection gelation and postoperative wound injection	Tang et al. (2019)
MC	One-pot synthesis	35–45°C	2 min	Reversible gel transition, high biological activity	Bone regeneration	Park et al. (2017)
MC-cTOCN-MMC	EDC/NHS-chemical coupling reaction	37°C	30 s	High drug loading, low cytotoxicity, resist adhesion, biodegradability, and excellent mechanical properties	Prevention of postoperative peritoneal adhesion	Sultana et al. (2020b)
CS/HPMC/Gl	Hydrophobic action	32–36°C	10 min	Good liquidity, biodegradability, low cytotoxicity, and slow-releasing potential	Biomedical field	Wang et al. (2016)
PF-127/HPMC	Hydrogen bonding	31.8–34.2°C	34.7–39.3 s	Excellent gel property, high adhesion, and sustained release	Treatment of postoperative eye inflammation	Morsi et al. (2016)

Moreover, various modified CS can also be used to prepare thermosensitive nanomaterials. For example, the isopropyl side chain introduced into the CS backbone to obtain a new modified CS. In fact, the existence of isopropyl side chains affects the solubility of the nanomaterial by altering its hydrophobicity. Therefore, the physical and chemical properties of modified CS can be adjusted by controlling the number of introduced hydrophobic side chains (Cok et al., 2020). It is reported that water-soluble CS derivatives containing glycol retain the characteristics of CS and are more suitable for biomedical field. Li et al. prepared a new injectable thermosensitive nanomaterial N-hexanoyl glycol CS (HGC) by exploiting the N-hexanoylation of glycol CS. The phase transition temperature of HGC is about 23–56°C, which depends on the level of acylation and polymer concentration. The thermosensitive nanomaterial is biodegradable, and the degree of degradation depends on the concentration of the polymer. Generally speaking, the nanomaterial exhibits thermoreversible sol–gel transition, long-term stability, and good biocompatibility (Li et al., 2018).

### 2.1.2 Methylcellulose

Methylcellulose (MC) is the simplest non-ionic cellulose ether, which is a cellulose derivative prepared by introducing methyl after the etherification reaction between cellulose and other chemical reagents. It exhibits unique temperature sensitivity. When the temperature is lower than LCST, MC forms hydrogen bonds with water molecules in the solution and shows a flowing viscous liquid. However, when the temperature is above LCST, the hydrogen bonds break, and -OCH_3_ is exposed to the solution, and the enhanced hydrophobic effect causes local aggregation and contraction of polymer molecular chains to form a cross-connected network structure, and the state changes from sol to gel (Kim et al., 2018a; Kim et al., 2018b).

The phase transition behavior of MC can be adjusted by molecular weight, degree of substitution, concentration, and the presence of salt (Shin et al., 2020). In the study of Park et al., the phase transition temperature of 8 wt% MC was about 35–45°C. The increase of MC concentration and the quantity of -OCH_3_ in MC solution lead to the increase in intermolecular and intramolecular interactions, thus reducing the gel temperature and gel time (Table 1) (Park et al. 2017). Because of the salting-out effect, the gelation rate of MC solution increased significantly. It is worth noting that MC solution containing sodium phosphate has the fastest gelation rate. The reason is that phosphate ions show a stronger salting-out effect than other ions, thus accelerating the hydrophobic interaction of MC molecules. Meanwhile, in solution, MC molecules containing phosphate ions form stable hydrogen bonds with water molecules. In summary, due to the existence of salting-out effect, the gel speed and mechanical properties of the MC gel are improved, while its injectability maintains an appropriate maximum force (Kim et al., 2018b). Moreover, Park et al. used the precursor salt of CaP to induce the salting-out effect and prepared a MC thermosensitive nanomaterial based on CaP nanoparticles by the one-pot method. In fact, CaP can reduce the formation of hydrogen bonds between polymers and water molecules, making water molecules more likely to react with the salt. This result promotes hydrophobic interactions between molecules, thus reducing the gel temperature and time. It can be seen that the stronger salting-out effect induces the stronger hydrophobic effect and gelation. Hence, the temperature and time of gelation rest with the properties of CaP precursor salt (Park et al., 2017). One year later, Kim et al. verified the injectability of the MC thermosensitive nanomaterial containing CaP nanoparticles. After adjusting the pH of the solution to the human physiological pH (7.4), the injection force of the nanomaterial did not exceed the upper limit of the injection force *in vivo* (30N). It can be seen that the nanomaterial has injectability suitable for injection in humans. In general, these studies provide effective methods for preparing injectable MC thermosensitive nanomaterials of CaP nanoparticles with biological activity (Kim et al., 2018a).

**FIGURE 2:**
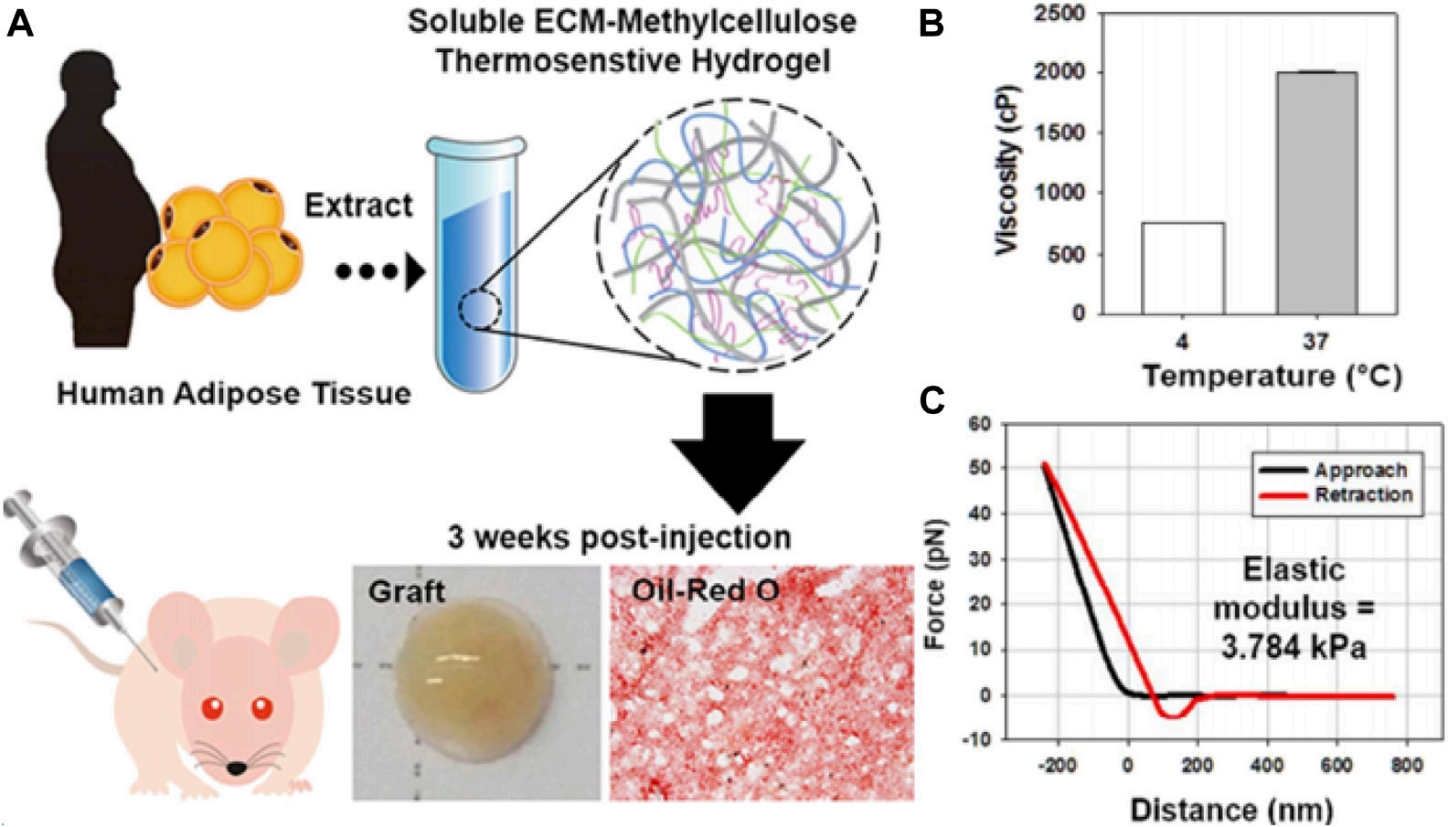
Representative studies of temperature-sensitive nanomaterials based on polysaccharide. **(A)** Cell-free hydrogel system based on a tissue-specific extracellular matrix for *in situ* adipose tissue regeneration. **(B)** Viscosity and **(C)** AFM-based force-distance curves of sECM-MC hydrogels. Reprinted with permission from Kim J. S. et al (2017). Copyright ^©^ 2017 American Chemical Society.

In 2019, Sultana et al. prepared a novel MC thermosensitive nanomaterial based on mitomycin C with modified tempo oxidized nanocellulose (TOCN), which also utilized hydrophobic interactions between methoxy-substituted MC molecules to achieve thermosensitive properties. It was found that the incorporation of MC promoted the formation of hydrogen bonds. The amount of MC is directly proportional to the gel time and gel strength, which is inversely proportional to the gel degradation rate. The optimized gel realizes the thermoreversible sol–gel transition at 37°C in only 30 s (Sultana et al., 2020b). The research team further optimized this system in 2020 and combined hyaluronic acid (HA) and polyethylene glycol (PEG) with MC and TOCN to prepare another thermoreversible nanomaterial, and further investigated the effect of different concentrations of HA on the gel system. Finally, it was concluded that the addition of HA can increase the adhesion resistance of the nanomaterial by adjusting the external construction, mechanical strength, gelation time, degradability, viscosity, and cell compatibility (Sultana et al., 2020a). Furthermore, Kim et al. reported a tissue-specific thermosensitive injectable nanomaterial (sECM-MC) consisted of an adipose-derived extracellular matrix and MC. At room temperature, sECM-MC is a lucid and slightly sticky solution. While at 37°C, the solution quickly forms a gel within 1 min. The stickiness of sECM-MC improved markedly from 752 to 2000 cp. In addition, the storage modulus (G′) of gel is about 3.8 kPa, and it was observed that sECM-MC nanomaterial has similar specificity for adipose tissue. Moreover, the sECM-MC nanomaterial likewise has excellent biocompatibility and injectability (Figure 2) (Kim J. S. et al., 2017).

### 2.1.3 Hydroxypropyl methylcellulose

Hydroxypropyl methylcellulose (HPMC) is a non-ionic cellulose ether, containing hydroxypropyl and methoxy side groups. It has unique thermal reversibility, which makes it an advantageous candidate for thermosensitive nanomaterials (Wang et al., 2016). In fact, as a hydrophilic polymer, HPMC has high swelling and surface activity. When HPMC contacts with water, the polymer undergoes chain relaxation and volume expansion, resulting in a thermoreversible sol–gel transition (Khanum et al., 2018).

According to reports, an injectable thermosensitive nanomaterial based on HPMC and CS is suitable for biomaterials. Based on this premise, Wang et al. prepared a novel thermosensitive nanomaterial with CS, HPMC, and glycerol as raw materials. Experiments show that the thermal gelation of the system conforms to the two-stage mechanism. Specifically, in the initial stage, the hydrophobic interactions between the polymers gradually increase with the destruction of the water sheath around the polysaccharide polymer, which has little effect on the modulus of gel. By further heating, hydrophobic regions of the polymer are connected to form a stable network structure, thus providing the gel with high elasticity, that is to say, the hydrophobic effect is the main driving force of the thermosensitive nanomaterial (Wang et al., 2016). Ghorpade et al. grafted β-CD onto HPMC by crosslinking the free carboxyl groups in β-CD and the hydroxyl group in HPMC to form ester bonds using citric acid as a crosslinker. The results show that the surface morphology of the nanomaterial depends on the crosslinking density and β-CD content. Meanwhile, the nanomaterial has a large swelling ratio, which is related to grafting amount, carboxyl content, and crosslinking degree (Ghorpade et al., 2016). By combining HPMC with pluronic (P407), a thermosensitive nanomaterial system with *in situ* gel can be obtained. The thermoreversible gelation behavior of the system is the result of micelle entanglement and accumulation. Subsequently, Wei et al. further studied the effect of thermosensitive nanomaterial composition on the gel temperature. The results show that the gel temperature decreases from 42 to 22°C with the increase of HPMC concentration. The reason can be explained by the dehydration of the HPMC molecule and the breaking of hydrogen bonds between water molecules and HPMC molecules (Wei et al., 2020a). However, to obtain the best gel and adhesive properties, Morsi et al. adopted a thermosensitive nanomaterial system, consisting of 14% PF-127/1.5% HPMC. The experimental results show that the nanomaterial has the best “++”grade gel, which shows a flowing sol state at 4°C and a non-flowing gel state at 35°C. In particular, if the concentrations of P407 and HPMC are reduced to 13 and 1%, respectively, the gelation ability will decrease, which may be attributed to insufficient polymer concentration to form a suitable three-dimensional micelle structure (Table 1) (Morsi et al., 2016). In addition, the binary polymer system containing P407 and HPMC exhibits excellent plasticity, thixotropy, and viscoelasticity (da Silva et al., 2020).

### 2.2 Amides

#### 2.2.1 Poly (N-isopropylacrylamide)

**FIGURE 3:**
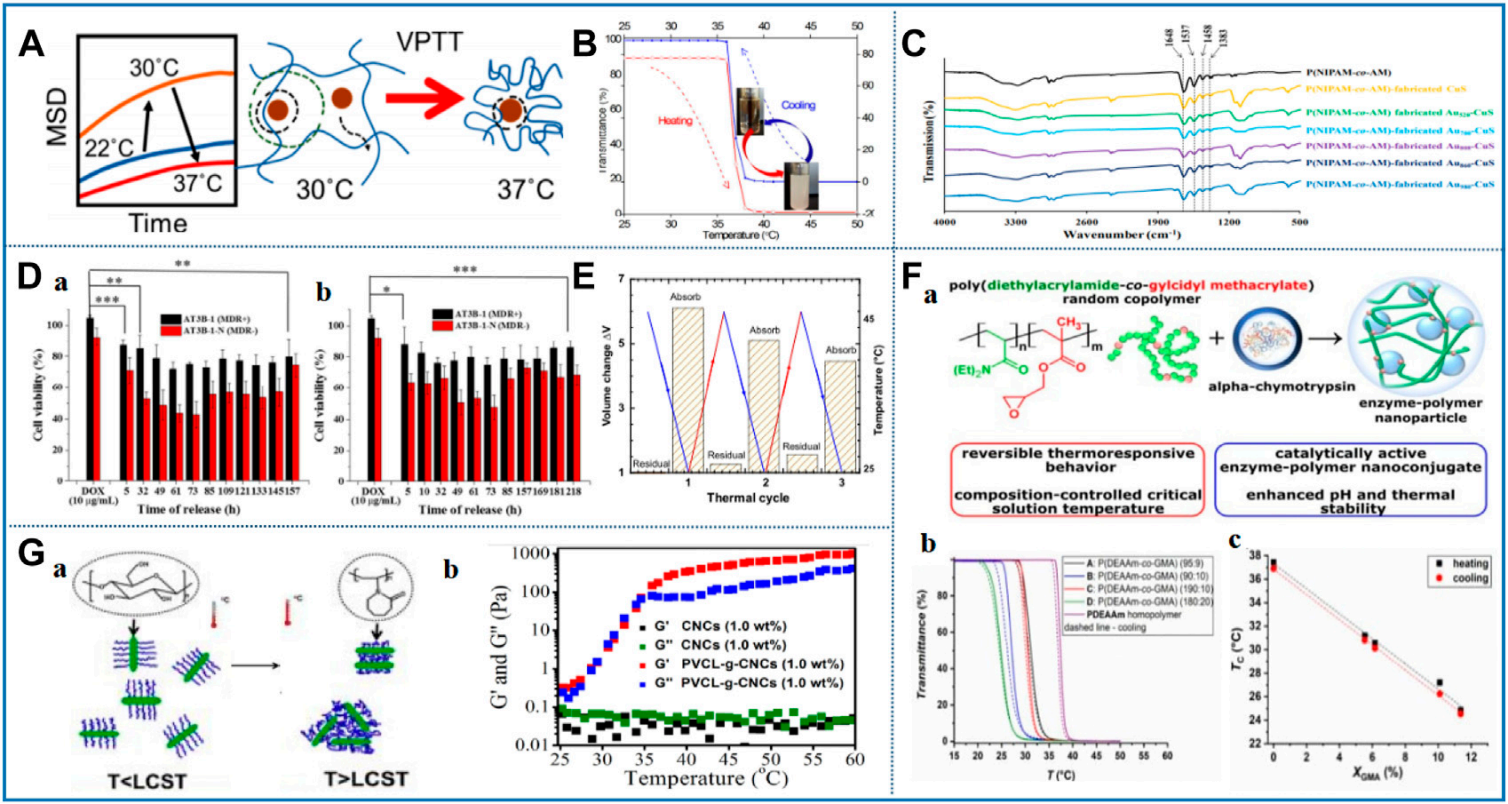
Representative studies of thermosensitive nanomaterials based on amide. **(A)** Temperature-dependent nanoparticle dynamics in poly (N-isopropylacrylamide) gels. Reprinted with permission from Parrish et al. (2018) Copyright ^©^ 2018 American Chemical Society **(B)** Temperature responsiveness of the three-component nanohybrid (Au@mSi@P). Reprinted with permission from Baek et al. (2016). Copyright ^©^ 2016 American Chemical Society **(C)** FTIR spectrum of P(NIPAM-co-AM) temperature-sensitive nanomaterial. Reprinted with permission from Chang et al. (2018). Copyright ^©^ 2018 American Chemical Society. **(D)** *In vitro* anticancer activities were evaluated using the MTT assays of thermoresponsive hydrogel formed by alginate-g-poly (N-isopropylacrylamide). Reprinted with permission from Liu M. et al (2017). Copyright ^©^ 2017 American Chemical Society **(E)** PNIPAm-POSS copolymer volume change during thermal cycles. Reprinted with permission from Romo-Uribe and Albanil 2019. Copyright ^©^ 2019 American Chemical Society. **(F)** (a) Synthesis of glycidyl-functional poly (N,N-diethylacrylamide-co-glycidyl methacrylate) copolymers *via* free radical copolymerization and the design of enzyme–polymer nanoconjugate with α-chymotrypsin: (b) thermoresponsive behavior of P (DEAAm-co-GMA) copolymers. Reprinted with permission from Kasza et al. (2021). Copyright ^©^ 2021 Polymers. **(G)** Elucidation of temperature responsive phase transition (a) and viscoelastic properties (G’and G″) (b) of PVCL-g-CNC copolymer influenced by temperature change. Reprinted with permission from Zhang et al. (2017). Copyright ^©^ 2017 American Chemical Society.

Poly (N-isopropylacrylamide) (PNIPAAm) is one of the most popular thermosensitive polymers. The LCST of PNIPAAm is 32–33°C, and it shows the transition from coil to ball above this temperature due to the change of the balance between hydrophilic and hydrophobic (Table 2) (Seo et al., 2014; Rajabi et al., 2021; Rizzo and Kehr, 2021). The macromolecular chain of PNIPAAm contains both hydrophobic isopropyl groups and hydrophilic amide groups. When the temperature is lower than LCST, PNIPAAm forms a hydrophilic polymer chain due to the hydrogen bond between amides and water molecules. However, when the temperature is higher than LCST, the amide group is dehydrated, such that the intermolecular hydrogen bond becomes weaker, and the hydrophobic interaction between the propyl group becomes stronger. Therefore, the polymer chain collapses into a small ball conformation to reduce the contact between hydrophobic groups and water (Seo et al., 2014; Parrish et al., 2018). Furthermore, the single particle tracking results of PNIPAAm showed that the gel underwent a dramatic transformation around the volume phase transition temperature (VPTT). Interestingly, polymer concentration and crosslinking density also affect VPTT. Increasing the concentration of a polymer or crosslinker results in a tighter gel structure and lower water retention capacity, resulting in swelling and a sharper VPTT. However, at higher polymer concentrations, the hydrophobic interaction is stronger, resulting in lower VPTT and it occurrs within a narrower temperature range (Figure 3A) (Parrish et al., 2018).

**TABLE 2:** List of thermosensitive nanomaterials based on Amide.

Thermosensitive nanomaterial	Synthetic mechanism	LCST	Superiority	Application field	Reference
p-NIBIm	Free radical polymerization	38–42°C	Extraordinary protein capture and release ability, low cytotoxicity	Drugs and the negatively charged molecules of DNA delivery system	Seo et al. (2014)
p (NIPAM-co-AM)	Ligand exchange method	39–41.5°C	Good dispersion, good biocompatibility, and reversible thermal response	Drug controlled release	Chang et al., 2018 Feng et al. (2019)
Alginate-g-PNIPAAm	Atom transfer radical polymerization	35°C	Good biocompatibility, biodegradability, low cytotoxicity, chelability, and easily chemically modified	Hydrophobic anticancer drugs of intelligent delivery system	Liu et al. (2017a)
HA-ss-PNIPAAm	Two-step amidation reaction	36.9°C	Size controllability, high tumor targeting, and low immunogenicity	Hydrophobic anticancer drug delivery	Chen et al. (2019a)
P (DEAAm-co- MBAAm-co-MAA)	Free radical polymerization	33.4°C	High porosity and hole interconnection	Tissue engineering scaffold	Horak et al. (2011)

Although PNIPAAm is the most popular thermosensitive polymers, its application has some limitations. For example, the LCST of PNIPAAm is 32–33°C, which is lower than the human body temperature. It also has the disadvantage of poor mechanical strength (Zhang K. et al., 2018). To solve the limitation of PNIPAAm, Baek et al. polymerized PNIPAAm with N-butyl imidazolium (BVIm) to improve its LCST. The results of thermal analysis showed that the LCST of thermosensitive nanomaterial p (NIPAAm-co-BVIm) increased from 32.3 to 40.85°C. This phenomenon is attributed to the repulsion of the positively charged BVIm in the copolymer chain, which may block the arrangement of the hydrophilic portion of BVIm with the hydrophobic portion of NIPAAm (Figure 3B) (Baek et al., 2016). Furthermore, Seo et al. obtained a novel thermosensitive nanomaterial (p-NIBIm) doped with ionic liquid by radical copolymerization of PNIPAAm and 1-butyl-3-vinyl imidazolium bromide ([BVIm]Br). However, compared with PNIPAAm, the zeta potential (+9.8 mV) and LCST value (38–42°C) of p-NIBIm were significantly increased (Seo et al., 2014). In 2019, Feng et al. polymerized NIPAAm and acrylamide by reversible addition-fragmentation chain transfer polymerization and synthesized thiol-terminated p (NIPAAm-co-AM). Through repeated experiments, it was found that when the molar ratio of NIPAAm to AM was 10:1, the LCST of the thermosensitive nanomaterial was 41.5°C (Table 2) (Feng et al., 2019). When the temperature is higher than LCST, the phase of the polymer changes from curl to collapse state. In addition, the Fourier transform infrared spectroscopy (FTIR) of p (NIPAAm-co-AM) showed obvious characteristic peaks at 1,648 cm^−1^ (C=O), 1,537 cm^−1^ (N-H), 1,458, and 1,383 cm^−1^ (C-H) (Figure 3C) (Chang et al., 2018). PNIPAAm combined with some salts can also adjust the LCST. For example, Alginate is a good choice for functional modification because of its excellent biocompatibility, biodegradability, non-toxicity, chelation ability, and facilitated chemical modification. Liu et al. prepared a thermally responsive nanomaterial (Alginate-g-PNIPAAm) by grafting PNIPAAm with Alginate. In addition, the nanomaterial was dissolved in water or PBS buffer solution at 25°C and formed self-assembled micelles with homogeneous size, good stability, and drug loading when the temperature increases above 37°C. As shown in Figure 3D, the results of *in vitro* cytotoxicity experiments show that the prepared Alginate-g-PNIPAAm has excellent biocompatibility and no side effects on normal tissues and cells. Therefore, it can be used as a drug carrier for drug delivery (Liu M. et al., 2017).

In view of the poor biodegradability of PNIPAAm, Chen et al. developed a thermosensitive nanomaterial (HA-ss-PNIPAAm) with excellent biocompatibility and biodegradability. In this scheme, HA and PNIPAAm were combined by disulfide bond in a two-step amidation reaction. Compared with PNIPAAm, the LCST of the solution increased from 32 to 36.9°C, which is closer to the human body temperature. It was found that the higher is the concentration of solution, the lower is the LCST (Chen J. et al., 2019). On this basis, the linear chain of PNIPAAm was grafted onto CS and further combined with HA to form a temperature-sensitive nanomaterial HA-CS-PNIPAAm. Its aqueous solution is flowable and injectable at room temperature, but turns into a gel at 30–32°C. It is important to note that the gel process of HA-CS-PNIPAAm is thermally reversible. At the same time, it is undeniable that the mechanical strength of HA-CS-PNIPAAm is higher than PNIPAAm, which is due to the addition of molecular weight and the entanglement of polymer chains (Chen et al., 2017). In 2019, Romo-Uribe et al. first reported the surprising double gel behavior with thermoplastic and thermal response of N-isopropyl acrylamide-polysiloxane oligomeric silsesquioxane nanomaterial (PNIPAAm-POSS). Here, the combination of water-insoluble POSS and hydrophilic PNIPAAm formed a physically crosslinked gel network. Interestingly, the physical gel showed a relatively high swelling rate, which exceeds the chemically crosslinked gel. This study showed that the PNIPAAm-POSS nanomaterial exhibited bidirectional shape memory behavior and self-healing behavior, which dissociated during shearing, but recovered immediately after the shearing stopped. In fact, the PNIPAAm-POSS nanomaterial opens opportunities for a new generation of intelligent materials, with bidirectional shape memory and self-repair ability (Figure 3E) (Romo-Uribe and Albanil, 2019).

#### 2.2.2 Poly (N, N-diethyl acrylamide)

Compared with widely used PNIPAAm, thermoresponsive poly (N, N-diethyl acrylamide) (PDEAAm) has better biocompatibility. Therefore, the research on PDEAAm and its derivatives has gained increasing attention in recent years (Kasza et al., 2021). Interestingly, its LCST value ranges from 25 to 36°C, approaching the physiological temperature of the human body. Its LCST can be adjusted by controlling the molecular weight, regularity, and concentration of the polymer (Işıklan and Kazan, 2018). When the temperature is lower than LCST, the polymer expands in water, but shrinks at the temperature above LCST. The expansion at low temperature is attributed to hydrogen bonds between amide groups of the polymer and water molecules. The breaking of hydrogen bonds and hydrophobic interactions become the dominant force, leading to the collapse of the gel structure at high temperature (Horak et al., 2011). Recently, Kasza et al. first reported a new reversible thermoresponsive nanomaterial p (DEAAm-co-GMA) synthesized by free radical copolymerization of DEAAm with glycidyl methacrylate (GMA). Among them, GMA is the most used functional monomer with epoxy side groups and can react with numerous nucleophiles through the ring-opening reaction. The results show that the LCST of p (DEAAm-co-GMA) is in the range of 24.8–37.4°C and decreases linearly with the decrease of DEAAm and with the increase of GMA content. In addition, the nanomaterial can also be coupled with protein to further improve its activity and stability (Figure 3F) (Kasza et al., 2021). It was reported that the hydrophobic properties of PDEAAm limit its application. Therefore, Işıklan et al. prepared a thermoresponsive nanomaterial (PVA-g-PDEAAM) grafted polyvinyl alcohol (PVA) with PDEAAm by microwave-assisted, and its LCST was 29–31°C. In this study, the results of differential scanning calorimetry (DSC) and thermogravimetric analysis (TGA) showed that the introduction of PDEAAm improved the thermal stability of PVA, and further confirmed the thermal responsiveness and biocompatibility of the synthesized nanomaterial (Işıklan and Kazan, 2018). In 2016, Yoon et al. synthesized an adjustable thermosensitive nanomaterial poly (N-acryloyl piperidine-co-N, N-diethyl acrylamide) with an LCST of 13°C. The LCST values of poly (N-acryloyl piperidine) and PDEAAm are 4 and 25°C, respectively. Thus, it can be seen that the LCST of the polymer can be indirectly adjusted by changing the ratio of the two monomers (Yoon et al., 2016). In addition, PDEAAm was also fabricated into a gel scaffold structure. Horak et al. synthesized PDEAAm gel scaffolds by free radical copolymerization of DEAAm, N, N-methylene bisacrylamide, and methacrylic acid in the presence of (NH_4_)_2_SO_4_ or NaCl. Among them, (NH_4_)_2_SO_4_ or NaCl acted as a porogen, and VPTT increased slightly with the increase in the porogen, and the average VPTT is 33.4°C. Therefore, the porogen have a significant impact on the formation of the gel network. In addition, the PDEAAm thermosensitive nanomaterial provides superior conditions due to its high porosity and pore interconnection and may become a useful tool in the field of tissue engineering (Horak et al., 2011).

#### 2.2.3 Poly (N-vinyl caprolactam)

Poly (N-vinyl caprolactam) (PNVCL) is a multifunctional polymer that exhibits thermal response behavior in water. Owing to its non-toxicity, compatibility with a variety of guest molecules (hydrophobic interactions and hydrogen bonding), and the phase transition temperature close to human body temperature, it is an attractive thermosensitive polymer (Siirilä et al., 2020; Halligan et al., 2017). It consists of repeating units composed of hydrophilic carboxyl groups and cyclic amides, where the amide group in the lactam ring is directly attached to the carbon–carbon skeleton (Siirilä et al., 2019). Similar to PNIPAAm, PNVCL also shows LCST at 32–34°C. Unlike PNIPAAm, PNVCL hydrolyzes and produces polymerized carboxylic acid under strong acidic conditions, rather than producing toxic amide compounds. Therefore, PNVCL is considered as a thermally responsive polymer with more biocompatibility (Indulekha et al., 2016). However, compared with the extensive research on thermosensitive nanomaterials based on PNIPAAm, the research on thermal response performance of PNVCL thermosensitive nanomaterials is still in its infancy (Zhang et al., 2017).

In the drug delivery system designed by Indulekha et al. hydrophilic CS was grafted onto PNVCL by the EDC/NHS coupling reaction. After comparison, it was found that the LCST of PNVCL after grafting with CS could be increased to 35°C, and its endothermic peak increased with the increase of CS concentration in grafting (Indulekha et al., 2016). Subsequently, Zhang et al. synthesized novel thermoresponsive nanomaterial PNVCL-grafted cellulose nanocrystals (PNVCL-g-CNCs) by surface initiated atom transfer radical polymerization. Rheological analysis shows that the G′ of PVCL-g-CNCs is greater than the loss modulus (G″) at 36°C. The phase transition mechanism is that hydrophilic PNVLC chain turns into a hydrophobic chain above LCST, and then collapses into a thin hydrophobic layer on the surface of CNCs. G′ and G″ increase significantly, which makes the LCST of PVCL-g-CNCs slightly higher than PNVCL (Figure 3G) (Zhang et al., 2017). In addition, the surface of PNVCL can be functionalized to make it undergo sol–gel phase transformation at higher LCST. Siirilä et al. added propyl acrylate to PNVCL. The addition of propyl acrylate successfully prepared the “clickable” nanomaterial with acetylene groups on the surface. Finally, two different glucosides and maltosides containing azide groups were grafted onto the surface of the nanomaterial. The nanomaterial with carbohydrate surface modification functionalization has high hydrophilicity and biocompatibility and can realize phase transformation at high temperature, which has the potential as a drug delivery system (Siirilä et al., 2020).

### 2.3 Polyester

Owing to their good biodegradability and biocompatibility, polylactic acid (PLA) and polycaprolactone (PCL) in aliphatic polyesters have been widely used as hydrophobic structural units of amphiphilic copolymers to prepare thermal responsive nanomaterials (Mao et al., 2018).

### 2.3.1 Polycaprolactone

In thermosensitive polymers, amphiphilic block copolymers can form thermosensitive nanomaterials by physical crosslinking, such as hydrophobic interactions (Buwalda et al., 2017). Various polyesters with hydrophobic properties serve as hydrophobic blocks, which can form thermosensitive nanomaterials with their complementary hydrophilic PEG. PCL, the most common polyester, has gradually become a research hotspot owing to its high crystallinity, hydrophobicity, biodegradability, and biocompatibility (Dabbaghi et al., 2021). It is a semi-crystalline polymer with a melting temperature (Tm) of 60°C and a glass transition temperature (Tg) of −60°C. It has good mechanical properties when stretched in the biaxial direction (Wang H. et al., 2017). According to reports, the temperature sensitive nanomaterial based on PEG/PCL amphiphilic block copolymer exhibits numerous advantages. On account of the existence of hydrophilic and hydrophobic phases, they can swell in water and organic media. Moreover, compared with hydrophilic nanomaterials, they have smaller water swelling and better mechanical properties. Compared with simple nanomaterials, they also have improved thermophysical property, structural stability, and adhesive ability (Dabbaghi et al., 2021).

**FIGURE 4:**
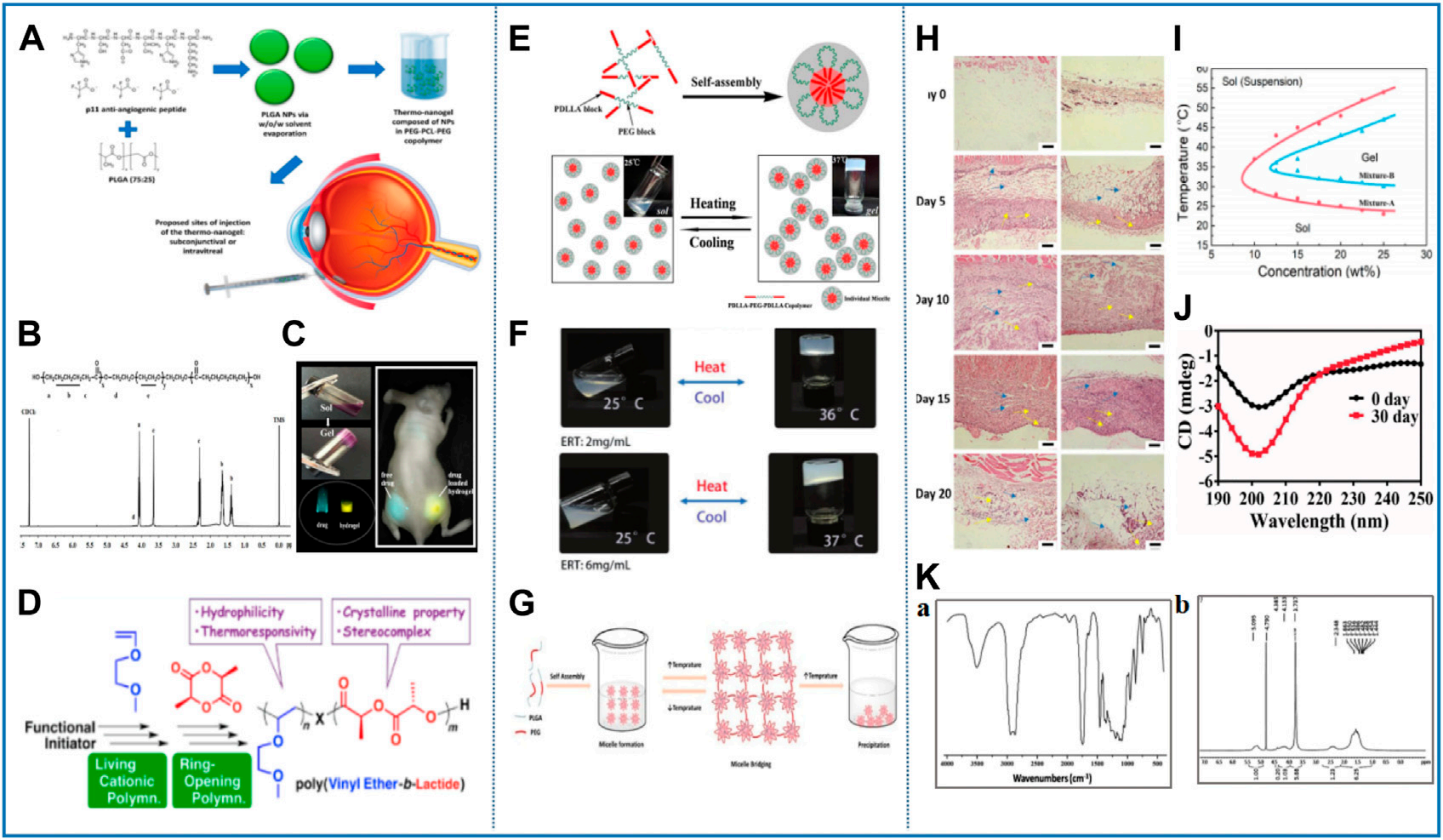
Representative studies of thermosensitive nanomaterials based on polyester. **(A)** Componential formulation and proposed delivery of the thermo-nanogel system. Reprinted with permission from du Toit et al. (2021). Copyright ^©^ 2021 Pharmaceutics. **(B)** Structure and ^1^H-NMR spectrum of PCEC copolymer. Reprinted with permission from Qu et al. (2018). Copyright ^©^ 2018 American Chemical Society. **(C)** Thermosensitive hydrogel with a protoporphyrin core based on a PEG and PCL copolymer (PCL-PEG-PPOR-PEG-PCL) as a potential visible biomedical implant. Reprinted with permission from Dong et al. (2016). Copyright ^©^ 2016 American Chemical Society. **(D)** New class of polylactide (PLA)-based block copolymers with thermoresponsive poly (vinyl ether) [poly (VE)] were precisely synthesized *via* successive living cationic polymerization of VE and ring-opening polymerization of lactide. Reprinted with permission from Seki et al. (2018). Copyright ^©^ 2018 American Chemical Society. **(E)** Schematic diagram of the process of temperature-induced physical gelation of the amphiphilic block copolymers. Reprinted with permission from Shi et al. (2016). Copyright ^©^ 2016 Scientific Reports. **(F)** Reversible sol–gel phase transition of ERT@HMSNs/gel composite. Reprinted with permission from Zhou et al. (2020). Copyright ^©^ Advanced Science. **(G)** Schematic diagram of the sol–gel transition of BAB type PLGA–PEG–PLGA triblock copolymer aqueous solution in response to temperature. Reprinted with permission from El-Zaafarany et al. (2018). Copyright ^©^ 2018 Pharmaceutics. **(H)** Storage stability of Col/Tra/Gel in 0 and 30 days at 4°C observed by the CD spectrogram. Reprinted with permission from Pan et al. (2018). Copyright ^©^ 2018 Drug Delivery. **(I)** H&E staining of surrounding tissues of PLGA–PEG–PLGA. Reprinted with permissio from Liu Y. et al (2017). Copyright ^©^ 2017 American Chemical Society. **(J)** Phase diagrams of the aqueous solutions of the copolymer mixtures with the indicated mix proportions. Reprinted with permissio from Chen X. et al (2019). Copyright ^©^ 2019 Theranostics. **(K)** FTIR (a) and ^1^H NMR (b) spectra of PCLA–PEG–PCLA triblock copolymer PCLA–PEG–PCLA. Reprinted with permission from Li T. et al (2020). Copyright ^©^ 2020 Drug Delivery.

As we all know, understanding the phase behavior is highly important for studying the physical properties and functions of thermosensitive nanomaterials (Sultana et al., 2020a). It has been shown that amphiphilic copolymers exhibit different thermally induced phase transitions in solution, depending on the property, molecular constitution, and molecular mass of the block (Feng et al., 2016; Mao et al., 2018). It is conceivable that the temperature sensitivity of PEG/PCL nanomaterials is also mainly dependent on its chemical constitution and the length and molecular mass of PCL units. For example, Perret et al. first combined hydrophobic PCL (A) and hydrophilic PEG (B) blocks in different proportions, resulting in linear AB biblock, BAB or ABA triblock, star block, multiblock, and grafted polymers (Deng et al., 2019; Dabbaghi et al., 2021). Unfortunately, as one of the most promising synthetic polymers, PCL shows inherent hydrophobicity, and it is difficult to degrade in aqueous solution. To improve the hydrophilicity of PCL, Wang et al. grafted the hydroxyl active site to the PCL chain by aminolysis and crosslinked PEG to the PCL chain through esterification to synthesize the PCL-PEG two-block polymer. From the static water contact angle test results, it is evident that the hydrophilicity of PEG-modified copolymer has been significantly improved (Wang H. et al., 2017). Meanwhile, Kondiah et al. developed a PEG-PCL-PEG (PECE) triblock copolymer with high hydrophilicity. The ^1^H NMR spectrum shows peaks at 3.35, 1.6, 2.2, and 3.92 ppm, which mainly originate from methylene protons of -(CH_2_)_3_-, -OCCH_2_-, and -CH_2_OC- in PCL blocks. The peak value of 3.65 can be attributed to the functional group of -CH_2_CO- in the PEG unit, and further confirmed that the ratio of PCL∶PEG was 1∶3.5 (Kondiah et al., 2017). Then, du Toit et al. studied the sol–gel transition of the PECE thermosensitive nanomaterial at different concentrations by the tube inversion method and rheological analysis. It was observed that the LCST decreased from 35 to 24°C and the UCST increased from 38 to 45°C when the concentration increased from 20 to 50% w/v. Obviously, with the increase of polymer concentration, the sol–gel transition window widens and the gel time gradually shortens (Figure 4A) (du Toit et al., 2021). In 2019, Lee et al. had a whimsy to add a group with an oxalate bond to the PECE thermosensitive nanomaterial to control the gel duration. The PEG–PCL–PEG triblock copolymer is transformed into the PEG–PCL diblock copolymer, with the degradation of the intermediate oxalate group, which exhibits different phase behaviors. By comparison, it was found that the gel window (34.6–37°C) of the triblock copolymer is wider, while the gel duration of diblock copolymer is shorter (Table 3) (Lee and Jeong, 2020). Inspired by this, Feng et al. successfully prepared P(CL-co-TOSUO)-PEG-P(CL-co-TOSUO) (PECT) triblock copolymers by introducing hydrophilic cycloether side groups 1,4,8-trioxa-[4.6]spiro-9-undecanone (TOSUO) into the main chain of PCL. The addition of the cycloether not only increases the hydrophilicity of PCL but also reduces its crystallinity, resulting in the decrease in gel strength. However, the gel modulus of PECT is only 50–70 Pa, which may limit its application. Subsequently, the group prepared another PEG-P(CL-co-TOSUO)-PEG (PECTE) thermosensitive nanomaterial by optimizing the hydrophilic/hydrophobic ratio and chemical composition of copolymer. The results of rheological analysis show that the gelation temperature of PECTE is 21°C, and PECTE has a relatively narrower gel window. By comparison, it was found that the G′ of PECTE is 100 times that of PECT, which markedly solved the problem of the low gel modulus. This fact also indicates that microstructure parameters (such as hydrophobic blocks and different chemical compositions) seriously affect the phase transition behavior (Feng et al., 2016). In addition, numerous groups have conducted more in-depth and extensive research works on PCL–PEG–PCL (PCEC) triblock thermosensitive nanomaterials. As shown in Figure 4B, the ^1^H NMR results of Qu et al. show that the characteristic peak at 3.65 ppm is the methylene proton in the PEG unit (CH_2_CH_2_O), the peaks at 2.32 and 4.07 ppm are methylene protons of -OCCH_2_- and -CH_2_OC- in the PCL unit, and the peaks at 1.32 and 1.56 ppm are repeated methylene proton peaks of PCL (Qu et al., 2018). Recently, compared with PECE, PCEC exhibited a lower critical gel temperature and wider gel window, which may be the result of greater aggregation due to bridging of PEG (Buwalda et al., 2017). To further enrich the application of PCEC, Dong et al. synthesized the multiblock thermosensitive nanomaterial (PCL–PEG–PPOR–PEG–PCL) by ring-opening polymerization with protoporphyrin (PPOR) as the core of the polymer skeleton. The copolymer exhibits high heat sensitivity and it takes only 1 minute for the transition from solution to gel at 37°C. However, when the temperature exceeds 41°C, G′ and G″ decrease rapidly, and the gel becomes a flowable sol. Compared with PCEC, the intervention of PPOR leads to some changes in the temperature sensitive properties of PCL–PEG–PPOR–PEG–PCL nanomaterial, but it did not significantly affect its thermosensitivity (Figure 4C) (Dong et al., 2016). Thus, the desired result can be achieved by modifying functional groups in the multiblock copolymer.

**TABLE 3:** List of thermosensitive nanomaterials based on polyester.

Thermosensitive nanomaterial	Synthetic mechanism	LCST	Gel Time	Superiority	Application field	Reference
PEG–PCL–PEG	Double emulsion method	32–45°C	--	Good biocompatibility and high hemolytic	Prevention of neovascularization, ophthalmic disease, and tumor treatment	Kondiah et al., 2017; du Toit et al., 2021; Lee and Jeong, (2020)
P(CL-co-TOSUO)- PEG-P(CL-co- TOSUO)	Ring-opening polymerization	24°C	--	Biodegradability, operability, good dispersibility, convenient preparation, and diversified degradation mechanisms	Drug delivery and tissue engineering	Feng et al. (2016)
PCL–PEG–PCL	Ring-opening polymerization	33°C	--	Good biocompatibility, support conditions for cell growth	Spinal fusion disease treatment	Qu et al. (2018)
PCL–PEG-–PPOR–PEG–PCL	Ring-opening polymerization	29–37°C	1 min	Injectability, good biocompatibility, and non-invasive	Adhesion prevention, drug and gene delivery, tissue regeneration, and visual biomedical implants	Dong et al. (2016)
PDLLA–PEG– PDLLA	Ring-opening polymerization	35–37°C	50–60 s	Injectability, reversible gel transition, low cytotoxicity, good inflammation, and reduce postoperative adhesion	Cancer treatment, sustained drug delivery, tissue regeneration, and wound dressings	Shi et al., 2016 Zhou et al. (2020)
CO_2_H-PDLLA-PEG-PDLLA-CO_2_H/NH_2_-PDLLA-PEG-NH_2_	Ring opening reaction, coupling reaction, and hydrogen bonding	32–39°C	<1 min	Good biocompatibility, biodegradability, excellent gel property, and mechanical strength	Combination therapy of tumor and drug delivery	Fentahun Darge et al. (2021)
PLGA–PEG–PLGA	Ring-opening polymerization	31–37°C	<1 min	Reversible gel transition, injectability, biodegradability, and low cytotoxicity	Protein/peptide drug delivery, local tumor therapy, and ophthalmic drug delivery	Shi et al., 2018 Liu et al. (2017b)
PCLA–PEG–PCLA	Ring-opening polymerization	37°C	--	Biodegradability, good biocompatibility, long persistence *in vivo*, low cytotoxicity, slight inflammatory reaction, and good biological stability	Intra-articular sustained administration and local drug release	Wang et al., 2017c Li et al. (2020b)

In addition to linear PEG/PCL copolymers, numerous star structures have also been used to prepare thermosensitivity nanomaterials. For example, Buwalda et al. synthesized and compared a series of linear and star PEG/PCL block copolymers. Among them, the star block copolymer showed better water solubility and formed a more uniform and transparent gel. If the ester bond between PEG and PCL is changed into an amide bond, the length of PCL blocks and the molecular weight are increased, which usually leads to a larger gel window, higher gel hardness, and enhanced stability *in vitro*. DLS analysis showed that low concentration linear and star PEG/PCL block copolymers can self-assemble in water to form micelles and aggregates with a hydrophobic PCL core and hydrophilic PEG shell (Buwalda et al., 2017). In general, in the amphiphilic block copolymers based on PEG/PCL, whether linear or star block polymers of AB, ABA, and BAB, the phase transition from sol to gel occurs with the increase of temperature in solution (Deng et al., 2019). The aforementioned studies show that the thermal sensitivity can be adjusted by the hydrophobic block length, polymer structure, and PEG–PCL connecting unit.

### 2.3.2 Polylactic acid

Because of its hydrophobicity, crystallinity, and good biocompatibility, polylactic acid (PLA) has attracted extensive research interest in materials, engineering, medicine, and other fields. Another important use of PLA is to form stereoscopic complexes as poly (L-lactide) (PLLA) and poly (D-lactide) (PDLA), which is used to enhance the mechanical and thermal properties of PLA (Figure 4D) (Seki et al., 2018). The research shows that poly (D,L-lactic acid) (PDLLA) is a common biodegradable polymer, and its degraded products are natural metabolites H_2_O and CO_2_, which can be further eliminated by the human body, so it shows excellent biocompatibility *in vivo* (Silva et al., 2017). These characteristics have stimulated the investigation of a series of copolymers composed of PLA and functional blocks to endow or optimize their properties (Seki et al., 2018). For instance, amphiphilic block copolymers consisting of PLA and PEG are the most convenient biomaterials for preparing thermosensitive nanomaterials. They self-assemble into micelles in aqueous solution, which aggregate to form a gel network with the increase in temperature. The gel behavior of these copolymers is mainly due to the dehydration of PEG at high temperature and the increase of micelle size (Fentahun Darge et al., 2021). In general, these amphiphilic block copolymers show obvious temperature sensitivity. This feature makes it feasible for PLA-based block copolymers to be used in various biomedical fields.

Previously, the amphiphilic triblock nanomaterial PDLLA–PEG–PDLLA (PLEL) has been reported in the literature. In 2016, Shi et al. first discovered and reported the reversible temperature-sensitive gelation behavior of the PLEL triblock nanomaterial. At low temperature, the nanomaterial exhibited an injectable sol state and transformed into a solid gel at 37°C, and then turned into a sol again with increasing temperature. No change was observed in the structure or properties of the nanomaterial during repeated sol–gel conversion. Although a slight lag was found between the heating and cooling curves, the changing trends of the transition temperature and G′ were almost similar, which further verified the thermal reversibility of physical gelation (Figure 4E) (Shi et al., 2016). To understand this phenomenon, Zheng et al. further studied the action principle of temperature sensitivity of amphiphilic block nanomaterials in water. The results show that when the temperature is lower than LCST, PLEL was self-assembled into micelle monomers by hydrophilic PEG shell and hydrophobic PDLLA core. When the temperature is increased to LCST, the aggregation and accumulation between micelles are enhanced, forming a stable gel network (Zheng et al., 2020; Hu et al., 2021). It can be seen that the sol–gel transition of the PLEL nanomaterial is related to the formation and subsequent entanglement of micelles. To quantitatively observe the gel behavior of PLEL, Zhou et al. conducted dynamic rheological analysis. Specifically, when the temperature is lower than 30°C, the G′ and G″ of gel are very low, indicating that the gel has good fluidity and injectability, and there is no risk of syringe blockage during injection. However, with the increase in temperature, G′ and G″ gradually increase until their values are equal, and at this time the transition from sol to gel occurs. As shown in Figure 4F, the gelation temperature of PLEL nanomaterial is approximately 35–37°C, which is near the body temperature (Zhou et al., 2020). To probe whether PLEL is biodegradable, Guo et al. monitored its degradation *in vitro* by gel permeation chromatography (GPC) and ^1^H NMR. The results showed that with the prolongation of degradation time at 37°C, the PLEL molecular weight was gradually reduced and the ethylene glycol component decreased, while the lactic acid component increased. The molecular weight only degraded by 20% after 30 days, which indicates that the PLEL copolymer has slow degradation behavior (Guo Z. et al., 2021). On the basis of the aforementioned studies, Fentahun Darge et al. modified PLEL with functional end groups to obtain two novel copolymers CO_2_H-PDLLA-PEG-PDLLA-CO_2_H and NH_2_-PDLLA-PEG-PDLLA-NH_2_, which were mixed together to improve the thermal gel properties and mechanical strength of the nanomaterial. In aqueous solution, the mixing of copolymers based on opposite charges can reduce the critical gel temperature to 32–39°C due to the formation of hydrogen bonds between carboxyl ends and amine ends. In addition, the micelles in the modified nanomaterial are more densely packed and the viscosity of the gel is increased (Table 3) (Fentahun Darge et al., 2021). In 2018, Seki et al. accurately synthesized a new PLA-based thermosensitivity nanomaterial by active cationic polymerization of vinyl ether and ring-opening polymerization of lactide. Interestingly, the thermal response behavior of this block nanomaterial depends heavily on the length of the PLA segment. When the PLA chain is short, the transition between micelles aggregation and phase separation is reversible, while when it is long, irreversible precipitation is observed. The permanent precipitation during heating is most likely due to the crystallization of longer PLA segments. In addition, the formation of the stereoscopic complexes between the PLLA and PDLA segments likewise affects the thermal response of the nanomaterial (Seki et al., 2018). These research data lay a good foundation for the development of new materials with unique properties.

#### 2.3.3 Poly (lactic acid–glycolic acid)

Poly (lactic acid–glycolic acid) (PLGA) copolymer is considered as one of the most widely used biodegradable polymers with biodegradability and biocompatibility and has been approved by regulatory agencies for human research and clinical use. In addition, PLGA can also be combined with other polymers or molecules for better biological properties (Mir et al., 2017; Martins et al., 2018). For example, the triblock thermosensitivity nanomaterial composed of PEG and PLGA undergoes a visible sol–gel transition at about 31°C. It also has the advantages of controllable properties, rapid synthesis, good reproducibility, and high yield, which further broadens its application range (Wang H. et al., 2017; Shang et al., 2017; Liu D. et al., 2019; Chen X. et al., 2019; Yang X. et al., 2020). Since body temperature can trigger clinical gelation, the temperature sensitive gel behavior of triblock copolymer provides a path for future smart nanomaterials (Liu Y. et al., 2017). In 2018, El-Zaafarany et al. synthesized and studied the PLGA–PEG–PLGA triblock copolymer. The copolymer is a yellow–brown semisolid with viscosity. The nuclear magnetic resonance spectrum shows that the characteristic peaks of -CH_3_ and -CH- of the lactide monomer are 1.6 and 5.2 ppm, respectively. In addition, the peak of -CH_2_- in the glycolide monomer at 4.8 ppm and the characteristic signal of methylene in the PEG monomer at 3.6 ppm. In fact, by analyzing the peak integral values related to lactide and glycolide, it can be determined that the chain number molecular weight of the copolymer is 5.2 kDa, and the molar ratio of lactide to glycolide is 3:1 (Figure 4G) (El-Zaafarany et al., 2018). At the same time, PLGA–PEG–PLGA also belongs to an amphiphilic block copolymer, where the PLGA block acts as a hydrophobic core, and the hydrophilic PEG block is wrapped around the periphery to form micelles. Then, with the increase in temperature, micelles further aggregate to form the gel (Jing et al., 2021). The analysis results of these items clearly showed the internal structural groups of PLGA–PEG–PLGA. Norouzi et al. pointed out that PLGA–PEG–PLGA has the characteristics of thermoreversible thermosensitive gel. When the temperature is lower than LCST, the nanomaterial is a transparent liquid with good injectability. When the temperature is higher than LCST, it becomes a white gel (Norouzi et al., 2021). Furthermore, Shi et al. proposed and further proved that the VPTT of the nanomaterial would increase as the growth of the length of the PEG, but decrease with the increase in the ratio of lactide to glycolide (Table 3) (Shi et al., 2018). Liu et al. also studied the G′ and G″ changes of thermal nanomaterials to assess the gel behavior of nanomaterials. The PLGA–PEG–PLGA thermosensitive nanomaterial exhibits a sol–gel transition with the increase of temperature, and the G′ and G″ reach the maximum value when approaching the physiological temperature (Liu D. et al., 2019). Even if the gel is formed at 37°C, its water content remains high (about 70wt%). In contrast, the ester bonds connected between PLGA–PEG–PLGA chains may be hydrolyzed, which affects the structure of the gel and leads to gel degradation. The *in vitro* degradation curve shows that it can be degraded and eliminated after treatment, thus avoiding surgical resection (Jin et al., 2020). In fact, the circular dichroism spectrum (CD spectrum) is a classic method to monitor the structural changes of biological macromolecules and evaluate biological activity. Pan et al. compared the CD spectra of PLGA–PEG–PLGA polymer solution between 0 and 30 days and found that the CD curve of the polymer changed significantly after 30 days of storage. The difference of the CD spectrum can be attributed to the degradation of PLGA–PEG–PLGA polymer (Figure 4H) (Pan et al., 2018). Liu et al. further show that PEG fragments were preferentially lost during the degradation process, while the PLGA content was relatively high. *In vivo*, PLGA fragments are degraded by anaerobic glucose metabolism. In contrast, PEG fragments are not biodegradable, and thus it will be eliminated by the kidney. Most importantly, the H&E stain of PLGA–PEG–PLGA injected into subcutaneous tissue showed no sign of tissue necrosis or edema during the whole implantation period, indicating that the nanomaterial has acceptable biocompatibility and is suitable for application *in vivo* (Figure 4I) (Liu Y. et al., 2017).

The PLGA–PEG–PLGA thermosensitive nanomaterial is popular on account of simple one-pot synthesis and good safety. However, the gel window is very narrow, which makes them unable to satisfy the diversified requirements of biomedical field (Chen X. et al., 2019; Yang X. et al., 2020). To compensate for such defects, it is necessary to improve and optimize the properties of thermosensitive nanomaterials, including sol–gel transition temperature, gel window width, and retention time. Wang et al. analyzed and pointed out that by physically modifying the sequence and ratio of lactide/glycolide, the length and molecular weight dispersion of PLGA or PEG block and the different end caps and stereochemical structures of the copolymer could affect the thermal sensitivity and related functions of the copolymer (Wang et al. 2017b). Inspired by this, Chen et al. constructed a PLGA–PEG–PLGA thermosensitive nanomaterial with adjustable gel performances by easily mixing two kinds of PLGA–PEG–PLGA with different molar ratios of PEG/PLGA. The results show that by simply adjusting the mixing ratio from 7:3 to 5:5, the Tg of the polymer increased from 23 to 31°C, which is a physiologically important temperature window (Figure 4J) (Chen X. et al., 2019). These methods effectively widen the temperature window of related polymers, which can meet the different requirements of biomedical applications. The knowledge of all the characteristics, potentials, and defects of PLGA, opens novel avenues for its application in the biomedical field (Martins et al., 2018). Multifunctional PLGA nanostructures provide a path for future nanomedicine, and it can simultaneously realize the real-time monitoring of drug delivery, molecular imaging, and therapeutic response (Mir et al., 2017).

### 2.3.4 Poly (ε-caprolactone-co-lactide)

Inspired by PLGA–PEG–PLGA, Wang et al. used poly (ε-caprolactone-co-lactide) (PCLA) with slow hydrolysis rate to replace the PLGA block in the copolymer. The lactone unit in it avoided the crystallization of the polymer in the sol state, thus making the application of PCLA–PEG–PCLA copolymer more extensive. Apart from the addition of PCL can reduce the acidic effect of degradation products compared with PLA alone. The stability is likewise improved (Wang et al., 2017c). Due to its good biodegradability, biocompatibility, and long *in vivo* persistence, the thermosensitive nanomaterial based on PCLA–PEG–PCLA have attracted the attention of Li et al. First, its chemical structure was studied by infrared spectroscopy. The terminal hydroxyl group was absorbed at 3,500 cm^−1^, and the peaks at 2,937 and 2,870 cm^−1^ were attributed to the stretching vibration of C-H. The stretching vibration of the C=O group shows strong absorption at 1,750 cm^−1^. The characteristic signals of the ^1^H NMR spectrum are consistent with the results of the infrared spectrum. Next, the sol–gel behavior of the nanomaterial was analyzed. The nanomaterial showed a flowing sol at 25°C, which was injectable, and quickly converted to an immobile gel at 37°C (Figure 4K) (Li T. et al., 2020). The rheological analysis results of van Midwoud et al. showed that the G′ of 25% gel (220 Pa at 40°C) is higher than that of 20% gel (166 Pa at 40°C). These data show that the concentration of the polymer is highly important to the gel strength. To explore the change of gel strength of PCLA–PEG–PCLA with different end-capping groups, the copolymers terminated by the acetyl and propyl groups were tested, respectively. Theoretically, the propyl-terminated copolymer will lead to the stronger hydrophobic interaction, thus forming a stronger gel (van Midwoud et al., 2018). As we all know, thermosensitive nanomaterials used *in vivo* should have good biocompatibility and should not trigger any immune reaction. Considering these characteristics, Phan et al. grafted bovine serum albumin (BSA) with PCLA to obtain a BSA/PCLA–PEG–PCLA bioconjugate. The use of protein-polymer bioconjugates endows thermosensitive nanomaterials with adjustable mechanical properties and easier chemical modification, thus producing intelligent polymer networks with precise functional properties. Owing to its special biological characteristics, including non-immunogenicity, bioadhesion and cell-to-cell interaction, bioadhesive nanomaterials show great potential in tissue repair (Phan et al., 2021). However, the existence of BSA requires strict experimental conditions, and the contamination of immunoglobulin and other plasma protein will affect the biological performance. Therefore, Duong et al. replaced BSA with HA to improve the security of conjugate. Notably, due to the strong temperature sensitivity of the PCLA–PEG–PCLA nanomaterial, the HA/PCLA–PEG–PCLA conjugate formed a stable gel under the skin and sustained external mechanical stress. Since this gel is formed by the self-assembly of condensed molecules, it shows biophysical and biochemical stability after being applied to warm-blooded animals (Duong et al., 2020). In 2021, Jung et al. used boric acid (BA) to modify the PCLA copolymer. The BA-PCLA copolymer exhibits a low critical gelation temperature at 34–37°C and can realize the phase transition from sol to gel between the room and body temperature. According to the phase diagram, the critical gel concentrations (CGC) of PCLA and BA-PCLA were determined to be 16.5 and 16 wt%, respectively. BA-PCLA with a concentration of 20 wt% showed a rapid sol–gel phase transition, and it was found that the formed gel was stable even after several hours of tilting. The results show that the PCLA nanomaterial can be used as injectable reservoirs for loading and controlling the release of chemotherapy drug (Jung et al., 2021).

### 2.4 Polyether

#### 2.4.1 Polyethylene glycol

Polyethylene glycol (PEG) has become the main component in the synthesized thermosensitive nanomaterials, owing to its advantages of good hydrophilicity, biocompatibility, biodegradability, non-toxicity, antigenicity, and immunogenicity (Wang et al., 2017b; Ding et al., 2017; Shi et al., 2021). For example, PEG can be copolymerized with PLA, PCL, PLLA, PLGA, and other polyesters to form copolymers with the AB diblock, ABA or BAB triblock, and star block structures (Wang et al., 2017b). However, the research based on PEG-polyester copolymer has been described in the previously mentioned polyester section, such that it will not be repeated here. This part mainly summarizes the biodegradable and thermosensitive nanoreactors modified by PEG.

**FIGURE 5:**
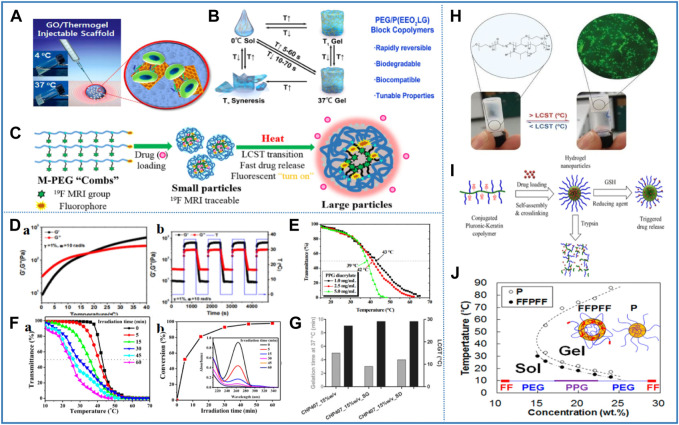
Representative studies of thermosensitive nanomaterials based on polyether. **(A)** Composite system of graphene oxide and polypeptide thermogel as an injectable 3D scaffold for adipogenic differentiation of tonsil-derived mesenchymal stem cells. Reprinted with permission from Patel et al. (2016). Copyright ^©^ 2016 American Chemical Society. **(B)** Temperature-dependent reversible sol−gel−sol transition of PEG/P (EEO_2_LG) hydrogels and the principal characteristics of the PEG/P (EEO_2_LG) hydrogels. Reprinted with permission from Zhao et al. (2021). Copyright ^©^ 2021 American Chemical Society. **(C)** Peptidic monodisperse PEG “combs” with fine-tunable LCST and multiple imaging modalities. Reprinted with permission from Zhu et al. (2019). Copyright ^©^ 2019 American Chemical Society. **(D)** Quantitative characterization of thermal sensitivity of injectable self-healing hydrogel with antimicrobial and antifouling properties. Reprinted with permission from Li et al. (2017). Copyright ^©^ 2017 American Chemical Society. **(E)** Transmittance curves of various concentrations of PPG diacrylate in water. Reprinted with permission from Cheng et al. (2017). Copyright ^©^ 2017 Polymer Chemistry. **(F)** (a) Transmittance change as a function of temperature for different concentrations of BU–PPG in aqueous solution. (b) Plot of LCST values vs. concentration of aqueous BU–PPG solution. Reprinted with permission from Gebeyehu et al. (2018). Copyright ^©^ 2018 American Chemical Society. **(G)** Tube inverting and gelation time tests allowed the investigation of the effects of particle loading on the gelation of CHP407-based sol–gel systems. Reprinted with permission from Pontremoli et al. (2018). Copyright ^©^ 2018 Chemical Engineering Journal. **(H)** Thermo-responsive poly (ε-caprolactone)-poly (ethylene/propylene glycol) copolymers as injectable hydrogels for cell therapies. Reprinted with permission from Brewer et al. (2020). Copyright ^©^ 2020 Polymers. **(I)** Novel hydrogel nanoparticles with dual triggerable release properties based on fibrous structural proteins (Keratin) and thermoresponsive copolymers (Pluronic). Reprinted with permission from Ghaffari et al. (2018). Copyright ^©^ 2018 American Chemical Society. **(J)** α,ω-diphenylalanine-end-capping of PEG–PPG–PEG polymers changes the micelle morphology and enhances stability of the thermogel. Reprinted with permission from Kim H. A. et al (2017). Copyright ^©^ 2017 American Chemical Society.

In fact, as early as 2016, Patel et al. synthesized a PEG–PA thermosensitive nanomaterial using N-carboxylic anhydride of L-alanine (PA). PEG–PA is unique in that it can be degraded by peptidases but is stable in the absence of peptidases. As a thermosensitive polymer, the nanomaterial has its general properties that the sol–gel transition of PEG–PA occurs with increasing temperature. Ideally, the thermosensitive polymer aqueous solution forms a gel *in situ* at 37°C and maintains the physical integrity of the 3D matrix (Figure 5A) (Patel et al., 2016). The thermosensitive nanomaterial based on peptides has also attracted the attention of Zhao et al. owing to its excellent biological properties and unique secondary conformation similar to natural peptides and proteins. They designed a series of block copolymers consisting of PEG and poly (γ-(2-(2-ethoxyethoxy)ethyl)-l-glutamate) (P (EEO_2_LG)) segments. The PEG/P (EEO_2_LG) block copolymer has a rapid thermal reversible sol–gel–sol transition on the range of a few seconds at 37°C and can be completely degraded *in vivo*. By comparison, it is found that the diblock copolymer has the strongest gelation ability, and the triblock copolymer the second. Although the gel window of the 4-arm star-shaped block copolymer is the narrowest, its gelation temperature is the most suitable for operation. In other words, both the P (EEO_2_LG) block length and PEG/P (EEO_2_LG) block ratio affect the secondary structure and the hydrophilic–hydrophobic equilibrium of the polymer, thus determining the gel behavior and properties of the nanomaterial (Figure 5B) (Zhao et al., 2021). Thermosensitive nanomaterials with adjustable LCST near body temperature hold high value in biomedicine. Therefore, to realize the fine tuning of LCST, Zhu et al. developed and designed M-PEG “comb” with fine tuning of LCST by accurately programming the structure of polypeptide monodisperse polyethylene glycol (M-PEG). Compared with the usual thermosensitive PEG block copolymers, the M-PEG “comb” has an accurate and programmable chemical structure, low molecular weight, and clear and fine adjustable LCST. By controlling the sizes of ω-amino acids and peptides in M-PEG, the LCST and biocompatibility can be finely adjusted. Notably, the flexibility of peptide synthesis will provide numerous potential functions for the M-PEG comb in biomedicine (Figure 5C) (Zhu et al., 2019). In addition, Li et al. used catechol functionalized PEG as the A block and poly{[2-(methacryloyloxy) ethyl] trimethyl ammonium iodide} as the B block to synthesize a self-healing nanomaterial based on the ABA triblock copolymer. Because A block is thermosensitive and B block is permanently hydrophilic, triblock copolymers can hydrate and exhibit liquid-like behavior at low temperature. However, the increase of temperature will lead to gelation. Among them, A block was dehydrated and combined into micelle nuclear crosslinking, and the middle B block acted as a network bridge, so that the nanomaterial shows excellent thermal sensitivity and thermal reversibility. Moreover, the nanomaterial also has excellent self-healing performance. The self-healing mechanism is mainly attributed to catechol-mediated hydrogen bonding and aromatic interactions (Figure 5D) (Li et al., 2017).

To improve the mechanical properties and biological activity of thermosensitive injectable nanomaterials, Chen et al. first modified the -CHO functional group to 4-arm polyethylene glycol (4-arm PEG) by esterification. Then the *in situ* crosslinked thermosensitive nanomaterial (GC/4-arm PEG-CHO) was prepared *via* Schiff base reaction. In the initial stage, the G″ of the nanomaterial is dominant. However, with the increase of temperature, the value of G′ began to increase rapidly and intersected with G″ at 60 s. This intersection point is the starting point of the gel in the chemical crosslinking system. After 600 s, G′ gradually reached stability, which indicated that the gelation process had been completed (Table 4) (Chen C. et al., 2020). In 2019, Tian et al. synthesized the 4-arm PEG–DA shape memory nanomaterial with dopamine (DA) as terminal, which exhibits rapid shape deformation ability induced by the body temperature and water and good mechanical properties. The advantage of the 4-arm PEG as the skeleton of the gel network is that it can obtain excellent tensile properties. Notably, the system can adjust the shape recovery temperature by flexibly controlling the water content of the gel and realize the shape memory induced by the body temperature. Specifically, with the increase of water content from 0 to 30 wt%, the Tm of the gel ranges from 51 to 25°C (Tian et al., 2019). Meanwhile, Ji et al. used another derivative of 4-arm PEG–4-arm PEG-NH_2_ and dextran oxide (ODEX) to prepare a thermosensitive nanomaterial. By analyzing the experimental data, it was found that the gel strength depended heavily on the mass ratio of 4-arm PEG-NH_2_ and ODEX. At higher mass ratio, G′ also increases. When the mass ratio is 1:1, the gel has higher G′ and the best adhesion effect (Ji et al., 2021). The understanding based on PEG thermosensitive nanomaterials has opened a new way for its future nanomedicine.

**TABLE 4:** List of thermosensitive nanomaterials based on polyether.

Thermosensitive nanomaterial	Synthetic mechanism	LCST	Gel Time	Superiority	Application field	Reference
GC/4-arm PEG-CHO	Schiff base reaction	37°C	60–600s	*In situ* crosslinking, good biocompatibility, and low cytotoxicity	Alternative scaffold for meniscus tissue engineering	Chen et al. (2020a)
GO/PEG–PA	Ring-opening polymerization	37°C	--	Mild gel forming condition	3D cell culture	Patel et al. (2016)
PEG/P(EEO_2_LG)	Ring-opening polymerization	37°C	10–70s	Rapid and reversible gel transition and good biocompatibility	3D cell culture	Zhao et al. (2021)
M-PEG	Solid phase peptide synthesis	10–62°C	--	Availability, good biocompatibility, versatility, adjustable LCST, and biodegradability	Slow controlled drug delivery and traceable imaging	Zhu et al. (2019)
PEG–PMETA–PEG	Reversible addition-fragmentation chain transfer polymerization	18°C	--	Reversible gel transition, self-healing and antimicrobial and antifouling properties	Implanted biomaterials and bioengineering	Li et al. (2017)
BA–PPG	One-step synthesis	33–56°C	--	Controllable physical property, simple manufacture, low cost, high efficiency, and versatility	Biomedical imaging and drug delivery	Cheng et al. (2017)
PEG–PPG–PEG	Nucleophilic substitution reaction	28–37°C	--	High hydrophilicity and good biological stability	Cell culture and long-term drug administration	Brewer et al. (2020)

### 2.4.2 Polypropylene glycol

Polypropylene glycol (PPG) is a water-soluble thermosensitive polymer with low molecular weight. When the temperature is as high as LCST, PPG will undergo a phase transition from hydrophilic to hydrophobic, and the molecular weight of the polymer will significantly affect LCST. In 2017, Cheng et al. first applied low molecular supramolecular polymers to thermosensitive nanomaterials. They designed an adenine-terminated bifunctional PPG (BA–PPG), and investigated the influence of the interaction of multiple hydrogen bonds in the oligomer structure of PPG on LCST. Interestingly, if the concentration of BA–PPG is increased from 1.0 to 5.0 mg/ml, the LCST is significantly reduced from 56 to 33°C. This indicates that the addition of the adenine group significantly changes the gel behavior of PPG in solution. Hence, the LCST of PPG can be easily adjusted by changing the concentration of BA–PPG, which provides an effective method for the formation of thermosensitive polymers with specific ideal properties (Figure 5E) (Cheng et al., 2017). In December of the same year, the team also reported another novel supramolecular polymer based on uracil (BU–PPG). The properties of this BU–PPG are similar to BA–PPG polymer, but they are not completely the same. Among them, uracil not only provides hydrogen bonds but also serves as a photosensitizer, providing this material with a unique temperature–light dual response. It is worth noting that the LCST of BU–PPG solution at a concentration of 4.0 mg/ml was 42°C, which is slightly higher than body temperature. This research provides a novel path for developing new supramolecular nanomaterials with multiple stimuli responses (Figure 5F) (Gebeyehu et al., 2018).

In addition, the triblock copolymer PEG–PPG–PEG (also known as Poloxamer or Pluronics), which consists of PPG central blocks and two PEG blocks, exhibits a temperature-induced reversible sol–gel transition at 37°C and is widely regarded as a potential candidate for various biological applications (Figure 5G) (Peng et al., 2016; Pontremoli et al., 2018; Qin et al., 2019). In particular, the PEG–PPG–PEG triblock copolymer has attracted significant attention because of its unique micellization behavior (Figure 5H) (Brewer et al., 2020). In detail, the PEG and PPG blocks hydrate at low temperature. When the temperature increases to the critical micelle temperature (CMT), the PPG block in the copolymer core and the PEG block in the shell undergo hydrophobic–hydrophilic interaction and self-assemble to form water-soluble micelles (Figure 5I) (Ghaffari et al., 2018; da Silva et al., 2020). Furthermore, at a constant concentration, the CMT of copolymer decreased with the increase of PPG ratio. This indicates that the polymerization and sol–gel behavior of Pluronics are the result of PPG incorporation (Brewer et al., 2020). Unfortunately, Pluronic exhibits poor mechanical properties, and its duration usually does not exceed 1 day *in vivo*. At the same time, the high CGC and non-degradability may lead to the accumulation of polymers *in vivo* and some side effects. These shortcomings limit the potential application of the Pluronic system, so people devoted themselves to modify the Pluronic copolymer (Shi et al., 2016; Shi et al., 2019). Because Pluronics itself is not biodegradable, it is necessary to introduce biodegradable parts to improve this application limitation. Therefore, Brewer et al. grafted PEG and PPG blocks onto the biodegradable PCL backbone by ring-opening polymerization. It was reported that the degree of PPG addition has a significant influence on the gelation and thermal response of the polymer, such that a series of PCL–PEG–PPG thermosensitive copolymers with different PEG:PPG ratios were synthesized. Although all copolymers showed thermal response characteristics, the copolymer with PEG:PPG ratio of 1:2 showed proper sol–gel transition at low concentration (10wt%). In addition, PCL-based copolymers are characterized by the presence of hydrolyzable and enzymatically degradable ester bonds, which make the materials easily degradable *in vivo* (Table 4) (Brewer et al., 2020). Indeed, as early as 2016, Wu et al. noticed a kind of polyester-poly [(R)-3-hydroxybutyrate] (PHB) with excellent biocompatibility and biodegradability, and then applied it to the thermosensitive nanomaterial, and designed the poly (PEG/PPG/PHB polyurethane) nanomaterial. The inner hydrophobic core composed of PPG and PHB blocks and the outer hydrophilic PEG chain constitute the structure of self-assembled micelles. Among them, the addition of PHB block remarkablely improved the hydrophobicity and mechanical properties of the gel and promoted the formation of gel. Surprisingly, the cell survival rate was still higher than 90% when the concentration of polymer reached 100 μg/ml. This shows that the thermosensitive nanomaterial has very low cytotoxicity and can be used safely *in vivo* (Wu et al., 2016). Two years later, Luo et al. found that although PHB was biodegradable, its brittleness and crystallization behavior still limited its wide application. To further adjust the mechanical properties of PHB, they replaced PHB in the system with poly [(R)-3-hydroxybutyrate-(R)-3-hydroxyhexanoate] (PHBHx) with higher elasticity and thermoplasticity. The amphiphilic copolymer in aqueous solution forms micelles at low concentrations. By discussing the influence of PHBHx segment on micelles, it was found that the synergistic effect of micelles is easier to be formed when the content of PHBHx and PPG is higher. This design may facilitate the construction of copolymers with biocompatibility, biodegradability, and low cytotoxicity (Luo et al., 2018). Nevertheless, short gel duration and low gel modulus are also disadvantages of Pluronic. In 2017, Kim et al. proposed that the aforementioned limitations of Pluronic can be overcome by selecting suitable conjugated groups. Here, they coupled Pluronics F127 (P) with diphenylalanine (FF) to prepare FF end cap F127 (FFPFF), in which the addition of FF can improve the mechanical properties and stability of the gel. In particular, the interference of end groups on micelle stacking is considered to be the reason for the narrowing of the gel window and the increase in sol–gel transition temperature (Figure 5J) (Kim H. A. et al., 2017).

## 3 Applications in biomedical field

The thermosensitive nanomaterial is considered as one of the most widely used biomaterials in biomedicine. Typically, the polymer solution is a flowing sol state at room temperature, but spontaneously becomes a non-flowing gel after responding to physiological temperature stimulation (Zhang et al., 2021). If drugs or cells are simply mixed into the sol and then injected into the target tissue, a static gel will be formed *in situ*. Because of this unique temperature sensitivity, thermosensitive nanomaterials based on thermosensitive polymers have been widely used in drug carriers (Guo et al., 2017b; Guo et al., 2021a; Guo et al., 2021b; Guo et al., 2021d), tissue engineering, and cell culture, sensing analysis (Guo et al., 2016a; Guo et al., 2016b; Liu F. et al., 2019; Guo et al., 2020b) (Shi et al., 2016; Shi et al., 2019).

### 3.1 Drug delivery

Thermosensitive nanomaterials are considered as a promising method to solve the problems and limitations of drug delivery and controlled release (Bordat et al., 2019). As an emerging drug carrier, nanomaterials based on thermosensitive polymer have been widely used to deliver drugs, cells, genes, protein, and other small molecule substances (Guo et al., 2020a; Guo Y. et al., 2022). This carrier can react according to the change of temperature, so that it can locate and control the drug release, which significantly improves the convenience and efficiency of drug delivery (Sun et al., 2020). It is a major breakthrough to change the pharmacokinetics and biological distribution of active pharmaceutical ingredients (API) by adding them into the nanomaterial, so as to prevent serious side effects and even improve the efficacy of the original API (Bordat et al., 2019). In addition, these nanocarriers can also be customized to target specific and exhibit delayed or controlled drug release by nanotechnology, (Karimi et al., 2016). It is worth mentioning that the thermosensitive nanomaterial exhibits good biocompatibility and biodegradability and has lower toxicity than the nanoparticle carrier (Sun et al., 2020).

**FIGURE 6:**
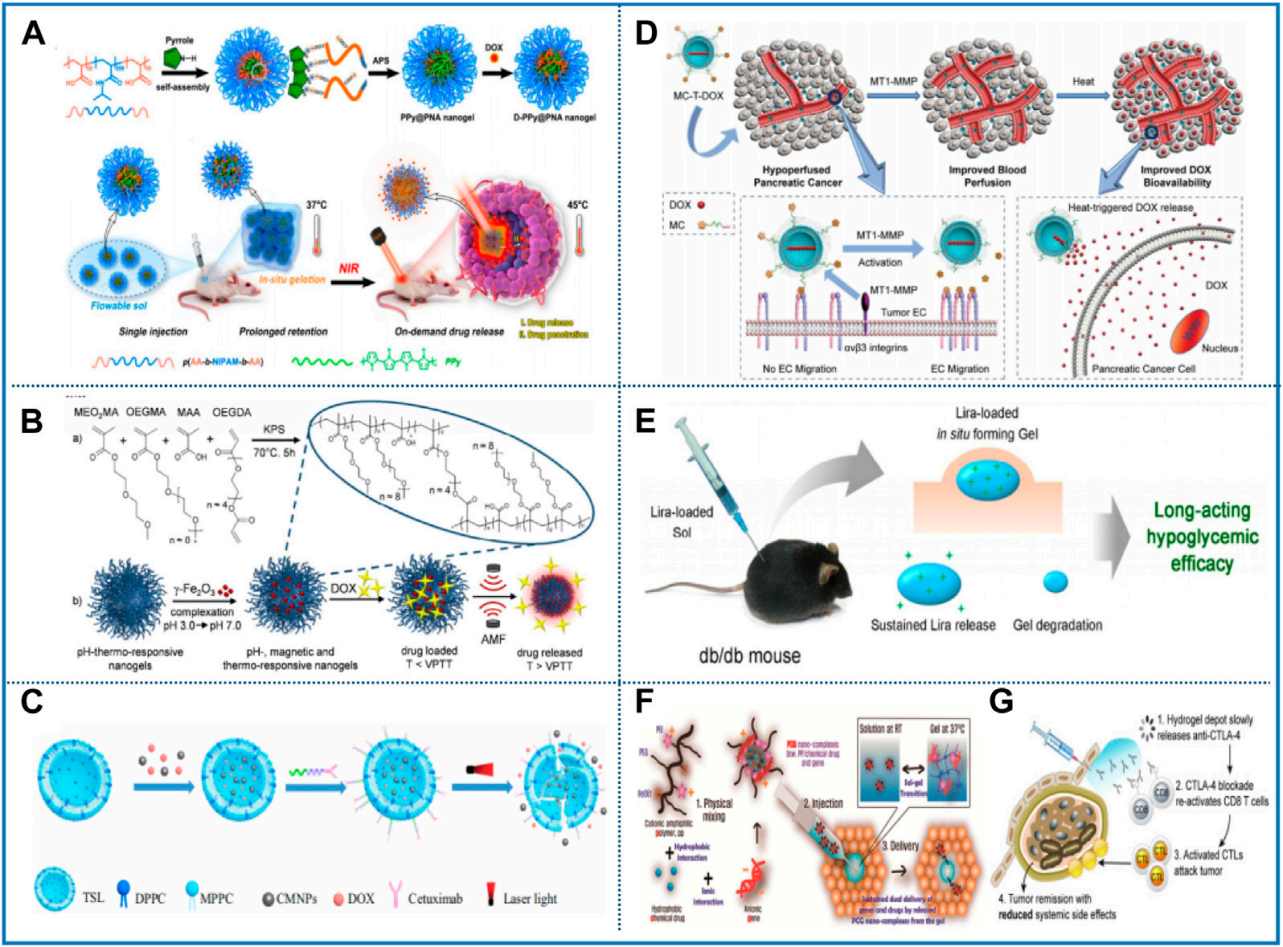
Representative studies of thermosensitive nanomaterials in drug delivery. **(A)** Schematic illustration of the preparation of D-PPy@PNA nanogels and D-PPy@PNA nanogels based *in situ* forming hydrogels for synergistic photothermal-chemotherapy. Reprinted with permission from Geng et al. (2020). Copyright ^©^ 2020 American Chemical Society. **(B)** Illustration of MagNanoGels synthesis and remotely controlled drug delivery under AMF. Reprinted with permission from Cazares-Cortes et al. (2017). Copyright ^©^ 2017 American Chemical Society. **(C)** Schematic illustration of NIR-triggered DOX release from CET-DOX-CMNP-TSLs. Reprinted with permission from Dorjsuren et al. (2020). Copyright ^©^ 2020 International Journal of Nanomedicine. **(D)** Mode of action by which MC-T-DOX effectively and specifically improves tumor blood perfusion and drug delivery in pancreatic cancer. Reprinted with permission from Wei et al. (2020b). Copyright ^©^ 2020 Advance Science. **(E)** Schematic illustration of the long-acting antidiabetic formulation using injectable hydrogel. Reprinted with permission from Chen et al. (2016). Copyright ^©^ 2016 American Chemical Society. **(F)** Injectable ternary nanocomplex hydrogel for long-term chemical drug/gene dual delivery. Reprinted with permission from Kim et al. (2016). Copyright ^©^ 2016 American Chemical Society. **(G)** Thermosensitive poloxamer 407 (P407) hydrogels were evaluated as slow release system for optimizing CTLA-4 therapy. Reprinted with permission from Chung et al. (2020). Copyright ^©^ 2020 Journal of Controlled Release.

In recent decades, multimodal combination therapy has attracted extensive attention from Geng et al. because of the synergistic effect of various antitumor mechanisms. They developed a new injectable drug delivery nanoplatform (D-PPy@PNAs) based on thermosensitive polypyrrole (PPy). The gel formed *in situ* shows ideal photothermal performance and thermosensitive sol–gel phase transition behavior. At the same time, the strong interaction between the carboxyl group in the PNA polymer and doxorubicin (DOX), as well as the π–π stacking interaction between the polypyrrole conjugated backbone and DOX, make D-PPy@PNAs have high drug loading and NIR controlled/sustained release behavior, which can realize the photothermal-chemotherapy synergism (Figure 6A) (Geng et al., 2020). To avoid the early release of drugs in the process of delivery, the targeted and controlled functions can be realized at the same time. Baek et al. developed a therapeutic diagnosis platform, in which gold nanorods coated with DOX were wrapped in a mesoporous silica nanoshell coated with thermosensitive p (NIPAAm-co-BVIm), which was used as the control switch of the temperature response. When the temperature was lower than LCST (40.85°C), hydrophilic groups in the polymer chain formed hydrogen bonds with water molecules and expanded, blocking the surface holes. However, above the LCST temperature, the polymer corona collapses due to the hydrogen bond breakage, so the mesopore gate is opened and the drug is released. Therefore, the temperature-dependent behavior of polymer shells is critical for on-demand drug release in nanodrug delivery systems (Baek et al., 2016). This is the most common combination therapy of photothermal therapy (PTT) and chemotherapy in cancer treatment. If the thermosensitive nanomaterial is complexed with magnetic nanoparticles, the long-distance release of drugs in cells can be triggered by the “hot spot” effect under the alternating magnetic field. The release amount of DOX is doubled. The enhanced drug release is caused by local heating that shrinks the polymer network. This nanoplatform opens a possible path for magnetocaloric therapy against cancer (Figure 6B) (Cazares-Cortes et al., 2017). In recent years, besides thermosensitive polymers, thermosensitive liposomes based on natural phase change materials (PCMs) have also been used as thermal response gating materials to control drug release. On this basis, Dai et al. encapsulated indocyanine green and DOX in liposomes and modified the surface with folic acid (FA) and gadolinium chelate (Gd). The intracellular transport mediated by the FA receptor on the surface showed good active tumor targeting ability (Guo et al., 2017a; Wang et al., 2018). The thermosensitive liposomal core exhibiting a sharp melting point at 39°C not only improves the biocompatibility of nanoparticles but also confers the function of on-demand drug release on nanoparticles. The results showed that the final release of DOX was 55% in the form of on–off irradiation within 100 min, which was much higher than without NIR irradiation. Multifunctional therapeutic nanoplatforms responsive to environmental stimuli are ideal for on-demand drug release (Dai et al., 2019). In addition, in the process of drug delivery, antibodies often play the role of targeting cancerous sites. Dorjsuren et al. investigated whether cetuximab (CET) could be coated on thermosensitive liposomes to realize the synergistic effect of targeted drug delivery and PTT, thus optimizing the therapeutic effect of breast cancer. Among them, CET coating can target the epidermal growth factor receptor (EGFR) overexpressed on the surface of cancer cells and realize the local PTT and the release of chemotherapy drugs under the irradiation of NIR, thus avoiding the toxic and side effects on normal tissues (Figure 6C) (Dorjsuren et al., 2020). Furthermore, the drug delivery system based on thermosensitive nanomaterials can be used not only for the treatment of common breast and lung cancer but also for other cancers. For instance, the DOX-loaded thermosensitive liposome (MC-T-DOX) constructed by Wei et al. It can retain most of the drugs in circulation and rapidly release drugs upon local heating of the tumor. Drug release curves showed that DOX release showed clear temperature dependence, with less than 10% of DOX released at 37°C but about 80% released at 42°C. These results indicated that heat-triggered release of MC-T-DOX in the tumor stroma significantly improved the delivery of DOX at the tumor site and resulted in more DOX uptake into tumor cells, thus effectively improving the bioavailability of DOX. MC-T-DOX significantly inhibited tumor growth in a mouse model of pancreatic cancer due to improved drug delivery (Figure 6D) (Wei et al., 2020b). Chen et al. prepared an injectable PEG/polyester thermosensitive nanomaterial for the administration of liralutide as a long-term antidiabetic system. Lira-containing polymer solutions form *in situ* drug reservoirs after subcutaneous injection, suggesting that they can be used as minimally invasive injectable vectors for sustained drug delivery. The release test *in vitro* showed that the drug-loading system exhibited sustained drug release for up to 10 days, and the cumulative release amount was 70%–80%. The hydrophobic interaction between the C16 side chain of Lira and the polyester segment of the carrier polymer leads to the sustained release of this drug. At the same time, the gel has a suitable degradation rate *in vivo*, which can meet the needs of repeated drug administration in diabetic patients (Figure 6E) (Chen et al., 2016).

Moreover, thermosensitive nanomaterials can also undertake the task of delivering other small molecular substances such as proteins, DNA, RNA, and antibiotics. In recent years, small interfering RNA (siRNA) therapy has shown great potential in disease treatment (Guo et al., 2021b; Cao et al., 2021). To promote the delivery of siRNA in cells, Fliervoet et al. developed an NPD thermosensitive nanomaterial composed of PNIPAAm, PEG, and poly (2-dimethylaminoethyl methacrylate) for the local and sustained release of siRNA. Compared with free siRNA, siRNA was loaded into a thermosensitive nanomaterial to achieve better control and sustained release. In this study, the release of siRNA was dependent on the dissolution of the hydrogel matrix rather than the degradation of the chemical crosslinks, which could provide an alternative strategy for tuning the release kinetics (Fliervoet et al., 2020). On this basis, Kim et al. combined gene therapy with chemotherapy to prepare a polymer/chemical drug/gene (PCG) composite thermosensitive nanomaterial for long-term dual delivery of anticancer drugs/siRNA. The nanomaterial undergoes sol–gel transition at 37°C and slowly releases PCG complexes. In addition, the release time of the PCG complex from the gel can be regulated by the concentration of the drug. The high concentration of DTX increases the hydrophobicity of the gel and avoids the contact of water molecules through hydrophobic condensation, resulting in the continuous separation of the PCG complex from the gel. This means that adjusting the concentration of hydrophobic drugs in the gel can affect the release time *in vivo* and the therapeutic window of the gel (Figure 6F) (Kim et al., 2016). Recently, Chung et al. first proposed poloxamer 407(P407) as a checkpoint antibody delivery system. The results showed that 25% P407 gel was injectable, and a reservoir of antibodies was rapidly established after subcutaneous injection by incorporation of antibodies into the gel by simple mixing. Subsequently, the antibody showed a low initial release and sustained release for up to 120 h *in vitro*. The bursting of the hydrogel and diffusion are the driving factors of IgG release (Figure 6G) (Chung et al., 2020). In a word, the drug delivery system based on thermosensitive nanomaterial has created a promising research approach for the optimization of cancer treatment.

### 3.2 Tissue engineering

Tissue engineering is a new medical field that provides a promising revolutionary strategy for the regeneration and repair of damaged organs or tissues. The construction of biomaterials that can imitate the characteristics of the extracellular matrix (ECM) of tissues, so as to control cell behavior and promote functional recovery of damaged tissues, mainly including skin, muscle, bone, nerve, vascular, and oral tissues (Curvello et al., 2019; Zhao et al., 2022). The thermosensitive nanomaterial is liquid at room temperature and exhibits a gel at body temperature, which can form various shapes according to the needs of defective parts. Therefore, it holds evident advantages as the carrier of cells and microtissues in the process of tissue healing (Orth et al., 2018).

Due to the avascular nature of cartilage, its inherent self-repairing ability is very poor. At present, there is no effective method to treat osteoarthritis caused by the damage and degeneration of articular cartilage. Therefore, Agas et al. developed a composite HA/PEG thermosensitive nanomaterial and it can reverse cartilage degradation. The system undergoes a phase transition at body temperature, producing a gel reservoir at the injection site. This minimally invasive drug delivery strategy not only inhibits the release of inflammatory molecules but also induces the formation of new cartilage. This study will provide a new idea for the application of thermosensitive nanomaterials in cartilage tissue engineering regeneration (Agas et al., 2019). Xu et al. provided another feasible strategy for cartilage repair that a cartilage regeneration scaffold *in vitro* based on the thermosensitive nanomaterial. *In vitro*, the chondrocytes of the goat auricle were encapsulated in a gel to form a chondrocyte-gel construct. The constructs were molded into different shapes of cartilage *in vitro*, which retained the original shape after implanted into the body and further matured to form homogeneous cartilage with their original shapes (Xu et al., 2020). Subsequently, Tang et al. built a multifunctional osteoarthritis treatment platform based on PLEL. The PLEL@PL-NPs thermosensitive nanomaterial is helpful for chondrocytes to resist inflammation and improve their ability of redifferentiation *in vitro* under the stimulation of interleukin-1β. In the treatment of osteoarthritis, it prevents the degeneration of early cartilage and promotes the repair of late cartilage (Tang et al., 2021). In recent years, microvessel fragments (MVFs) that can release angiogenic growth factors have been introduced into regenerative medicine. Orth et al. suggested that applying MVF to bone defects may be a promising novel strategy to promote bone healing. The bone defect of mice was filled with a thermosensitive nanomaterial mixed with MVF. Finally, the results showed that the application of MVF-loaded nanomaterial in the bone healing model of rats significantly improves angiogenesis and bone repair (Orth et al., 2018).

**FIGURE 7:**
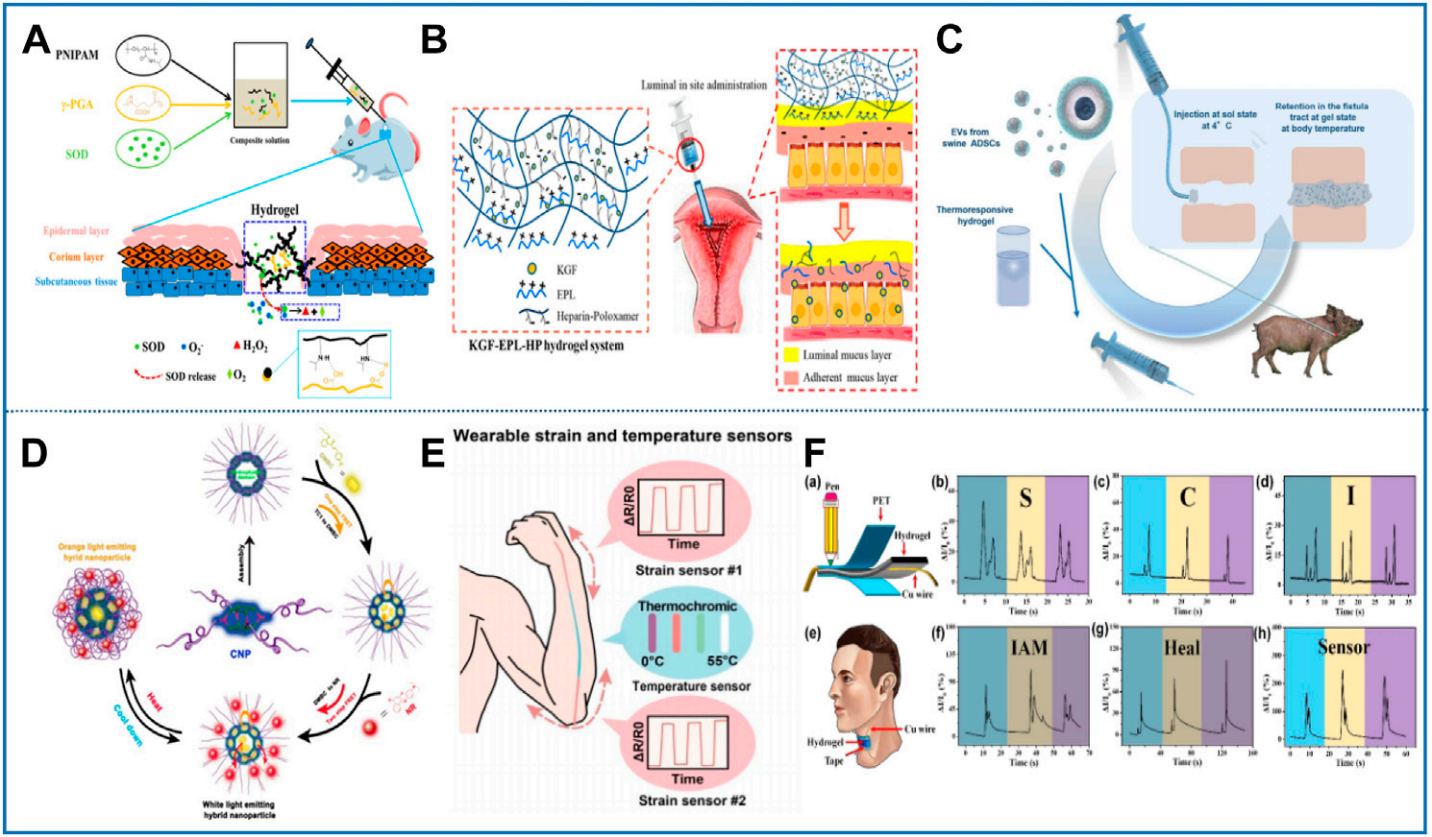
Representative studies of thermosensitive nanomaterials in tissue engineering and sensing analysis. **(A)** Poly (N-Isopropyl-acrylamide)/poly (γ-glutamic acid) thermo-sensitive hydrogels loaded with superoxide dismutase for wound dressing application. Reprinted with permission from Dong et al. (2020). Copyright ^©^ 2020 International Journal of Nanomedicine. **(B)** Scheme of thermo-sensitive bioadhesive KGF-EPL-HP hydrogel for injured uterus. Reprinted with permission from Xu et al. (2017). Copyright ^©^ 2017 American Chemical Society. **(C)** Thermoresponsive hydrogel gelling *in situ* at body temperature was administered locally into the fistula tract of a pig. Reprinted with permission from Silva et al. (2018). Copyright ^©^ 2018 American Chemical Society. **(D)** Graphical representation of CNP assembles into hybrid nanoparticle. Reprinted with permission from Wang et al. (2020). Copyright ^©^ 2019 American Chemical Society. **(E)** Multifunctional conductive hydrogel/thermochromic elastomer hybrid fibers with core–shell segmental configuration for wearable strain and temperature sensors. Reprinted with permission from Chen J. et al (2020). Copyright ^©^ 2020 American Chemical Society. **(F)** PPBN-hydrogel sensor for a flexible touch keyboard and wearable phonatory-recognition platform. Reprinted with permission from Ge et al. (2020). Copyright ^©^ 2019 American Chemical Society.

Thermosensitive nanomaterials have the characteristics of promoting cell adhesion and proliferation, which show great potential in promoting various wound healing (Zhang Y. et al., 2018). Dong et al. made use of this characteristic to prepare a wound dressing, which can repair chronic wounds. Notably, this thermosensitive nanomaterial combines the advantages of superoxide dismutase, PNIPAAm, and poly (γ-glutamic acid) to reduce oxidative stress, endow heat sensitivity, and create a humid microenvironment, which is beneficial for the healing of chronic wounds (Figure 7A) (Dong et al., 2020). In addition, Xu et al. designed a mucosal adhesion delivery system to promote wound healing of damaged endometrium using a thermosensitive nanomaterial. This system can enhance the retention and absorption of keratinocyte growth factor in the uterine cavity. After treatment, the morphology of the injured endometrium was well repaired. Surprisingly, not only the proliferation of endometrial epithelial cells and glands is evidently enhanced but also the angiogenesis in the regenerating endometrium (Figure 7B) (Xu et al., 2017). In 2018, Silva et al. designed extracellular vesicles (EVs) as the next generation of bioregenerative nanotherapy agents for esophageal fistula healing. EVs derived from adipose stem cells (ADSCs) were locally administered in the porcine fistula model by Pluronics F127. The thermosensitive nanomaterial gels rapidly at body temperature, and it retains EVs in the entire fistula and prevents intravenous injection from being washed by fistula secretions. More importantly, EVs can significantly reduce fibrosis, reduce inflammation, and promote angiogenesis (Guo et al., 2021c). In short, the use of the thermosensitive nanomaterial to transport EVs brings a prospect for the management of postoperative fistulas (Figure 7C) (Silva et al., 2018). Xu et al. prepared a thermosensitive nanomaterial that can continuously release aspirin and erythropoietin using CS/β-GP/gelatin. It is worth noting that the thermosensitive nanomaterial can fill the tiny tissue gaps caused by periodontitis by simple injection, thus playing the synergistic role of anti-inflammatory and tissue regeneration, which provides a novel path for the clinical treatment of periodontitis (Xu et al., 2019).

### 3.3 Sensing analysis

Currently, the sensors based on thermosensitive polymers have obvious advantages in biosensing analysis because of excellent flexibility, extensibility, and toughness. Here, Büning et al. first proposed a biosensor capable of detecting K^+^ concentration *in vivo*. Through the copolymerization of PNIPAAm with hydrophilic monomer, the K^+^ concentration can be accurately measured at physiological temperature. Surprisingly, the composite thermosensitive biosensor showed good sensing performance for K^+^ (about 5 mM) and low cross sensitivity for Na^+^ (about 125 mM). Therefore, this strategy makes it possible for future biosensors or implants to be applied to patients with hyperkalemia (Büning et al., 2018). Wang et al. (2020) developed a fluorescent probe with good cell compatibility, readability, and high resolution in living cells. The fluorescent probe shows a reversible response to temperature stimulation and could detect the intracellular temperature through the change of fluorescent color. It is used as a probe for temperature sensing in living cells with a temperature resolution of at least 0.5°C (Figure 7D). With the development of science and technology, flexible wearable sensors have gradually entered the public’s vision. Aiming at the problem of single functionality, Chen et al. reported a multifunctional wearable strain and temperature sensor to monitor human movement and ambient temperature (Figure 7E) (Chen J. et al., 2020). In 2019, Ge et al. proposed a thermosensitive nanosensor with self-healing, long-lasting heat resistance, and dual sensing. The thermosensitive sensor shows high sensitivity, high strain coefficient, wide sensing strain range, low detection limit, and high signal-to-noise ratio. Based on its excellent mechanical reception and thermal sensitivity, it can realize a “heating indicator” for human forehead temperature detection, which shows promising practical application in wearable devices (Figure 7F) (Ge et al., 2020). In the following year, Yang et al. endowed the thermosensitive nanosensor with shape memory and frost resistance. Among them, the addition of CS provides the nanosensor with electrical conductivity, such that it can perfectly detect the tiny motion of the human body. In addition, the orientation of PVA molecular chain under external force and the orientation after heating are used as a thermal response shape memory sensor. Glycerol significantly improved the frost resistance of the gel (Yang T. et al., 2020).

As the basic units of life, biomolecules play a crucial role in many biological processes in the body. Therefore, thermosensitive biosensors for the detection of small molecules in living organisms have gradually come into view in recent years. Among them, the detection of cancer- and tumor-related markers has attracted extensive attention. For example, Liu et al. designed and synthesized an miRNA-responsive vision and thermosensitive probe consisting of the DNA hydrogel coated with horseradish peroxidase (HRP). In the presence of miRNA, substrate DNA adapter strands hybridize with miRNA, resulting in hydrogel collapse and HRP release. Then the miRNA was detected indirectly according to a series of changes caused by the released HRP. The sensor has a wide linear range (0.5–4.0 µm) and a detection limit of 7.8 nM. It has the advantages of low cost, easy operation, and high sensitivity and has great potential in biomolecular detection and biomedical applications (Liu et al., 2021b). Second, abnormal expression of a DNA methyltransferase (MTase) content is also a biomarker for predicting cancer. To this end, Niu et al. developed a biosensor based on the thermosensitive polymer PNIPAAm to measure DNA methylation and MTase M. SssI activity. Experimental data showed that the detection limit of the sensor was as low as 0.18 U/ml, which indicated that the method had high sensitivity and selectivity for the detection of DNA methylation and M. SssI MTase activity. This highly sensitive biosensor can be used as a useful tool in diagnostic and biomedical research (Niu et al., 2018). In 2020, Li et al. first used manganese acetylacetone (III) as a single precursor to synthesize manganese oxide-doped CDs (MnOx-CDs). Based on the interaction between MnOx-CDs and Fe^3+^, the fluorescence probe of Fe^3+^ was proven to have a linear range of 0.02–0.6 mM. In addition, the quenched fluorescence can also be restored after dissociation of the MnOx-CDs-Fe^3+^ complex by biothiols. Therefore, the continuous, selective, and sensitive detection of Fe^3+^ and biothiol can be achieved simultaneously. In addition, MnOx-CDs have reversible thermosensitive fluorescence properties *in vitro* ranging from 10 to 60°C and can be used as a thermometer in living cells. This provides the possibility to explore manganese oxide-doped CD in future biomedical applications (Li P. et al., 2020).

In fact, the unique thermal behavior of the thermosensitive nanomaterial can reduce the interference of the background, thus improving the detection sensitivity. Hence, it is also deeply appreciated by researchers in the field of electrochemical sensors. In 2019, Mutharani et al. prepared an electrochemical sensor for temperature-controlled smart switches to detect 5-fluorouracil (5-FU), which consists of thermosensitive PNIPAAm and conductive poly (3-ethylenedioxythiophene) Compared with 25°C (0.37 μm), the lower limit of detection was 15 nM at 40°C. The sensor has high sensitivity and reproducibility, which realizes the temperature switch control of 5-FU for the first time (Mutharani et al., 2020). In 2018, Chen et al. developed a thermosensitivity electrochemical sensor using poly (2-(2-methoxyethoxy)ethyl methacrylate) (PMEO_2_MA) to detect ractopamine, an animal growth promoter harmful to the human body. The research shows that when the temperature is higher than the LCST of PMEO_2_MA, a larger oxidation peak current can be observed. This peak disappears at lower temperatures. The sensor detects ractopamine in a range of 0.1–3.1 μm, with a detection limit of 82 nM. Therefore, the sensor has high sensitivity and reproducibility and can be widely used in the determination of ractopamine in pork samples (Chen et al., 2018). It was reported that PEO-b-P(NIPAAm-co-SHMA), another derivative of PNIPAAm, has excellent properties that are sensitive to Al^3+^, Zn^2+^, and the temperature. This novel multifunctional fluorescent chemosensor selectively recognizes Al^3+^ and Zn^2+^, according to the difference of fluorescence-response time and fluorescence color. More specifically, at high temperature, it can significantly improve the detection sensitivity by forming a hydrophobic region within the thermally induced region (Han et al., 2017).

### 3.4 Cell culture

**FIGURE 8:**
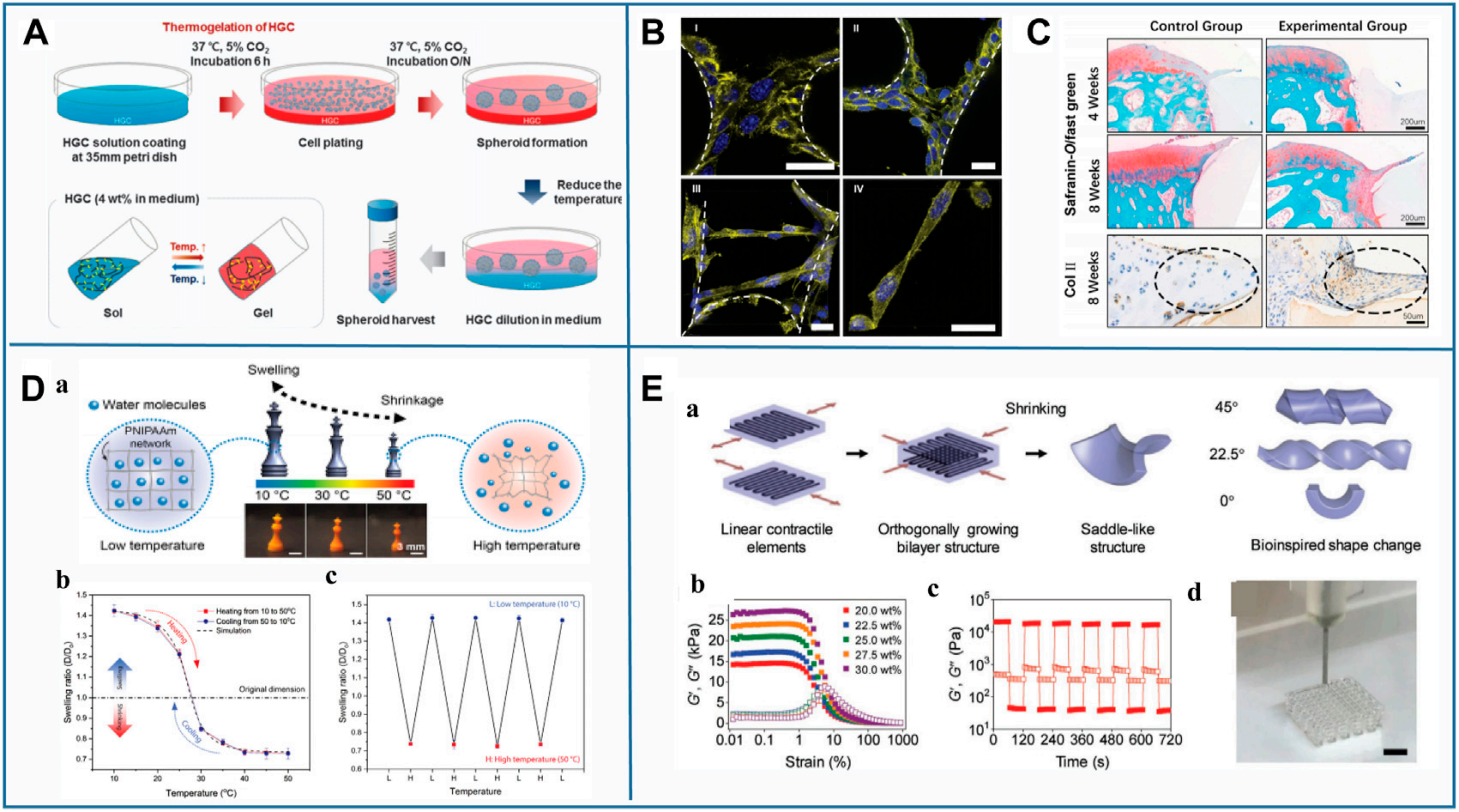
Representative studies of thermosensitive nanomaterials in cell culture and 3D printing. **(A)** Schematic illustration of coating the surface of a cell culture plate using HGC and the process of spheroid formation on an HGC-coated plate. Reprinted with permission from Cho et al. (2016). Copyright ^©^ 2016 NPG Asia Materials. **(B)** 3D fibroblast growth inside interlinked macroporous microgel scaffolds. Reprinted with permission from Rommel et al. (2022). Copyright ^©^ 2022 Advance Science. **(C)** Histological analysis of cartilage regeneration. Reprinted with permission from Guo J. et al (2022). Copyright ^©^ 2022 American Chemical Society. **(D)** Temperature responsive swelling of 3D printed PNIPAAm hydrogel structure (b) and (c) reversible temperature-dependent swelling/shrinkage of PNIPAAm hydrogel. Reprinted with permission from Han et al. (2018). Copyright ^©^ 2018 Scientific Reports. **(E)** Characterization of 3D printing of orthogonally growing bilayer structures of hydrogels with programmed motions. Reprinted with permissio from Arslan et al. (2019) Copyright ^©^ 2018 Advance Science.

Cultivating cells in culture medium is the most common way to cultivate cells. However, researchers gradually realize that the cell state in a 2D culture environment is not exactly the same as that *in vivo*. To better realize the study of physiological and pathological activities of cells *in vitro*, 3D cell culture has gradually developed, which aimed at simulating the *in vivo* environment (Sun et al., 2021). In fact, as early as 2016, there was interest in thermosensitive nanomaterials for 3D cell culture. For example, the carboxymethyl chitin thermosensitive nanomaterial proposed by Liu et al. is soft and malleable, and its G′ is comparable to type I collagen, matrix glue, and soft tissue, which is close to the cellular environment *in vivo*. It was observed that the 3D-crosslinked porous structure can facilitate the transport of nutrients, CO_2_ and O_2_, and cell migration, which is beneficial for cell growth and proliferation (Liu et al., 2016). In 2019, Bi et al. developed a similar 3D cell culture biomaterial based on thermosensitive furanyl-modified hydroxypropyl chitin. The results of 3D cell culture showed that the double crosslinked nanomaterial coated with HeLa cells formed *in situ*, which has the ability of continuous proliferation and self-assembly to form 3D multicellular spheroids (Bi et al., 2019). However, Cho et al. reported a novel thermosensitive nanomaterial N-acylated glycol chitosan for culturing cardiomyocytes *in vitro*. When it is applied to the surface coating of a cell Petri dish, multicellular spheres can be formed quickly and efficiently, and size of spheres varies with the cell density. In addition, the system can maintain its cellular function for a long time. More importantly, the system can be used for co-culture of heterotypic cells to form spheres that mimic biological systems (Figure 8A) (Cho et al., 2016). In 2021, a novel 3D macroporous construction was created by Rommel et al. to realize 3D cell culture *in vitro*, which can produce macroporous structures with pores up to hundreds of microns. This structure provides an open and friendly environment for cells with variable structure, biochemical clues, and mechanical characteristics to create a 3D cell network. In particular, the increase in the number of cells in macropores enhances the communication between cells, which is highly important for numerous physiological processes (Figure 8B) (Rommel et al., 2022).

Among many cell cultures, the cultivation of pluripotent stem cells is crucial for biomedical applications. Based on this, Chang et al. established a 3D cell culture system of human-induced pluripotent stem cells (hiPSCs). The original/primed pluripotency of hiPSCs was altered by culturing hiPSCs on CS. In this system, CS not only maintains the proliferation and pluripotency of hiPSCs but also converts the activated hiPSCs to the original state (Chang et al., 2021). The collected human ADSCs were mixed with CS/β-GP/Col nanomaterials and cultured *in vitro* at 37°C. Song et al. found that the novel nanomaterial provided a good culture environment for ADSCs, and the morphology, activity, and proliferation of the cells are well maintained. In addition, *in vivo* research results show that ADSCs-loaded nanomaterials can differentiate into adipose tissue, indicating good histocompatibility and fat formation ability (Song et al., 2017). Qu et al. investigated 3D cell culture substrates for another stem cell, bone mesenchymal stem cells (BMSCs). After culturing BMSCs in Arg-Gly-Asp (RGD)-modified hydroxybutyl chitosan (HBC) nanomaterials (HBC-RGD) for 7 days, it was found that the number of cells in HBC-RGD was higher than that in HBC. This result indicates that the 3D environment of the HBC-RGD nanomaterial is more favorable for the proliferation and adhesion of BMSCs (Qu et al., 2019). In summary, with the continuous development of life sciences, the demand for 3D cell culture technology is increasing. The superiorities of the 3D cell culture system established by encapsulating cells in thermosensitive nanomaterials are irreplaceable by 2D cell culture. Hence, 3D cell culture will gradually replace 2D cell culture, which significantly promotes the applications in biomedicine (Sun et al., 2021).

### 3.5 3D printing

With the rapid development of biology and materials science, as a novel biological manufacturing technology, 3D printing has entered a new stage of development (Rajabi et al., 2021). 3D printing can accurately realize multiple materials and scales manufacturing and optimize the manufacturing of biomaterials with complex composition and structure (Li C. et al., 2020). Therefore, 3D printing ink or other 3D printing scaffold coatings represented by thermosensitive nanomaterials have great prospects in the development of next-generation biomedical implants (Rajabi et al., 2021). However, in the cell load structure that accurately controls spatial relationships, the main bottleneck of bioprinting is the lack of biocompatible inks and printing conditions. To this end, Roehm et al. used thermosensitive CS-gelatin (CG) as a novel bioprinting ink to obtain 3D, sterile, cell-filled structures using an inexpensive bioprinter. The evaluation results indicate the possibility of using CG to print cell-loaded structures and the applicability of novel bioinks (Roehm and Madihally, 2017). However, Liu et al. developed a new biocompatible bioink composed of functional CS, HA, and matrix glue. The bioink rapidly gels in 20 s and has the ability to spontaneous covalent crosslinking, which can help to fabricate neural tissue scaffolds that mimic natural spinal white matter by means of a 3D printing strategy. The fabricated scaffolds enable optimal localization of neural stem cells and can provide favorable microenvironmental and biochemical cues for cell–cell and cell–matrix interactions (Liu et al., 2021a). Heiden et al. reported that the cooling time of gelatin-based bioinks can be maintained within a few seconds (<10 s), thus allowing printing into dimensionally stable complex objects. In this study, the thermoreversibility of the thermosensitive nanomaterial allows it to be recycled repeatedly during subsequent printing processes. More reprint cycles can be achieved by restoring printability by rehydration of the biogel and lowering the printing temperature or by increasing the printing speed and reducing the heating time (Heiden et al., 2022). Chae et al. built 3D constructs using tissue-specific ECM components and stem cells using 3D cell printing technology. Among them, the bioink used for 3D cell printing should have certain rheological properties to ensure shape fidelity and printability, and a series of rheological tests have also verified this characteristic. Different tissue structures were then fabricated using this innovative bioengineering strategy (Chae et al., 2022).

On the other hand, the lack of effective preparation method of printable ink and weak mechanical properties hinder the development and application of functional 3D printing thermosensitive nanomaterials. Here, Guo proposed a mild pre-crosslinking method to make the loosely crosslinked cellulose network by self-assembly to achieve desirable mechanical properties and printability. Predictably, due to its high flexibility and excellent mechanical properties for the construction of diverse structures, it can stimulate the development of other bioinks and thermosensitive nanomaterials with powerful 3D printing capabilities, which will eventually be popularized and applied in different fields (Figure 8C) (Guo J. et al., 2022). Han et al. reported on the 3D printed structure of PNIPAAm. Control of the temperature-dependent deformation of the 3D printed PNIPAAm is attained by changing the fabrication process parameters and the polymer resin composition. It is also demonstrated that the continuous deformation of 3D printed PNIPAAm structures can be achieved by selectively introducing ionic monomers to change the swelling transition temperature of PNIPAAm. Therefore, the 3D printing method with fast, high-resolution, and temperature response has unlimited development prospects in various fields (Figure 8D) (Han et al., 2018). In 2018, Arslan conceived of a 3D printing method based on gel phase inks with shear-thinning properties. In this work, the PNIPAAm ink is in the gel phase with excellent shear thinning and recovery properties, which are crucial for 3D printing. In fact, fast recovery seriously affects high-resolution 3D printing, as it prevents the ink from flowing after extrusion (Figure 8E) (Arslan et al., 2019). All in all, the bioink based on thermosensitive nanomaterials expands the range of available bioinks and opens many opportunities for biomedical applications, such as tissue engineering and soft robotics (Fang et al., 2021).

## 4 Conclusion and future perspectives

In recent years, with the increase of novel intelligent polymer materials and nanotechnology, thermosensitive polymers have become the focus of research in the biomedical field. Among them, the thermosensitive nanomaterial based on thermosensitive polymer has attracted increasing attention owing to its unique low toxicity, good biocompatibility, biodegradability, and injectability. It not only plays an important role in drug delivery but also holds great significance in tissue sensing and biomimetic medicine. According to statistics, the future development of thermosensitive nanomaterials may be applied to the treatment of chronic diseases requiring routine dose administration, such as diabetes and cancer multimode therapy. The combination of the temperature sensitive nanomaterial with other multi reactive nanomaterials with different stimuli (light, pH, enzyme, mechanical force, *etc*.) can improve the efficiency of diagnosis and treatment. The design of stimulus-sensitive nanomaterials may be attempted according to the final demand to achieve appropriate functions. So far, the utilization rate of the UCST polymer remains low. The future research direction may be used to study nanomaterials that can provide flexibility for designing new thermal response platforms for biomedical applications.

## Glossary

LCST: low critical solution temperature
UCST: high critical solution temperature
CS: chitosan
β-GP: β-glycerophosphate
Col: collagen
HPC: hydroxypropyl cellulose
G′: storage modulus
G′′: loss modulus
β-CD: β-cyclodextrin
HGC: N-hydroxymethyl chitosan
MC: methylcellulose
TOCN: tempo-oxidized nanocellulose
HA: hyaluronic acid
PEG: polyethylene glycol
HPMC: hydroxypropyl methylcellulose
P407/PF127: pluronic
PNIPAAm: poly (N-isopropylacrylamide)
VPTT: volume phase transition temperature
BVIm: N-butyl imidazolium
FTIR: Fourier transform infrared spectroscopy
PDEAAm: poly (N, N-diethyl acrylamide)
GMA: epoxypropyl methacrylate
PVA: polyvinyl alcohol
DSC: differential scanning calorimetry
TGA: thermogravimetric analysis
PNVCL: poly (N-vinyl caprolactam)
PLA: polylactic acid
PCL: polycaprolactone
TOSUO: 1,4,8-trioxa-[4.6]spiro-9-undecanone
Tm: melting temperature
Tg: glass transition temperature
PECE: PEG–PCL–PEG
PECTE: PEG-P(CL-co-TOSUO)-PEG
PCEC: PCL–PEG–PCL
PECT: P(CL-co-TOSUO)-PEG-P(CL-co-TOSUO)
PPOR: protoporphyrin
PLLA: poly (L-lactide)
PDLA: poly (D-lactide)
PDLLA: poly (D, L-lactic acid)
PLEL: PDLLA-PEG-PDLLA
GPC: gel permeation chromatography
CGC: critical gel concentrations
CD spectrum: circular dichroism spectrum
PLGA: poly (lactic acid-glycolic acid)
PCLA: poly (ε-caprolactone-co-lactide)
PCM: phase change materials
BSA: bovine serum albumin
BA: boric acid
4-arm PEG: 4-arm polyethylene glycol
DA: dopamine
HRP: horseradish peroxidase
ODEX: dextran oxide
PA: L-alanine
(P(EEO_2_LG): poly(γ-(2-(2-ethoxyethoxy)ethyl)-l-glutamate)
M-PEG: monodisperse polyethylene glycol
PPG: polypropylene glycol
CMT: critical micelle temperature
PHB: poly[(R)-3-hydroxybutyrate]
PHBHx: poly[(R)-3-hydroxybutyrate-(R)-3-hydroxyhexanoate]
API: active pharmaceutical ingredients
PPy: polypyrrole
DOX: doxorubicin
PTT: photothermal therapy
FA: folic acid
Gd: gadolinium chelate
EGFR: epidermal growth factor receptor
CET: cetuximab
siRNA: small interfering RNA
PCG: polymer/chemical drug/gene
ECM: extracellular matrix
BMSCs: bone mesenchymal stem cells
MVF: microvessel fragments
HBC: hydroxybutyl chitosan
EVs: extracellular vesicles
ADSCs: adipose stem cells
5- FU: 5-fluorouracil
PMEO_2_MA: poly(2-(2-methoxyethoxy)ethyl methacrylate)
hiPSCs: human induced pluripotent stem cells
CG: CS-gelatin
